# Evolution of Cell Wall Polymers in Tip-Growing Land Plant Gametophytes: Composition, Distribution, Functional Aspects and Their Remodeling

**DOI:** 10.3389/fpls.2019.00441

**Published:** 2019-04-18

**Authors:** Jérémy Dehors, Alain Mareck, Marie-Christine Kiefer-Meyer, Laurence Menu-Bouaouiche, Arnaud Lehner, Jean-Claude Mollet

**Affiliations:** Normandie Univ, UNIROUEN, Laboratoire de Glycobiologie et Matrice Extracellulaire Végétale, Rouen, France

**Keywords:** cell-wall, gametophyte, tip-growth, pollen-tube, protonema, rhizoid, evolution, land plants

## Abstract

During evolution of land plants, the first colonizing species presented leafy-dominant gametophytes, found in non-vascular plants (bryophytes). Today, bryophytes include liverworts, mosses, and hornworts. In the first seedless vascular plants (lycophytes), the sporophytic stage of life started to be predominant. In the seed producing plants, gymnosperms and angiosperms , the gametophytic stage is restricted to reproduction. In mosses and ferns, the haploid spores germinate and form a protonema, which develops into a leafy gametophyte producing rhizoids for anchorage, water and nutrient uptakes. The basal gymnosperms (cycads and *Ginkgo*) reproduce by zooidogamy. Their pollen grains develop a multi-branched pollen tube that penetrates the nucellus and releases flagellated sperm cells that swim to the egg cell. The pollen grain of other gymnosperms (conifers and gnetophytes) as well as angiosperms germinates and produces a pollen tube that directly delivers the sperm cells to the ovule (siphonogamy). These different gametophytes, which are short or long-lived structures, share a common tip-growing mode of cell expansion. Tip-growth requires a massive cell wall deposition to promote cell elongation, but also a tight spatial and temporal control of the cell wall remodeling in order to modulate the mechanical properties of the cell wall. The growth rate of these cells is very variable depending on the structure and the species, ranging from very slow (protonemata, rhizoids, and some gymnosperm pollen tubes), to a slow to fast-growth in other gymnosperms and angiosperms. In addition, the structural diversity of the female counterparts in angiosperms (dry, semi-dry *vs* wet stigmas, short *vs* long, solid *vs* hollow styles) will impact the speed and efficiency of sperm delivery. As the evolution and diversity of the cell wall polysaccharides accompanied the diversification of cell wall structural proteins and remodeling enzymes, this review focuses on our current knowledge on the biochemistry, the distribution and remodeling of the main cell wall polymers (including cellulose, hemicelluloses, pectins, callose, arabinogalactan-proteins and extensins), during the tip-expansion of gametophytes from bryophytes, pteridophytes (lycophytes and monilophytes), gymnosperms and the monocot and eudicot angiosperms.

## 1. Introduction

**Figure 1:**
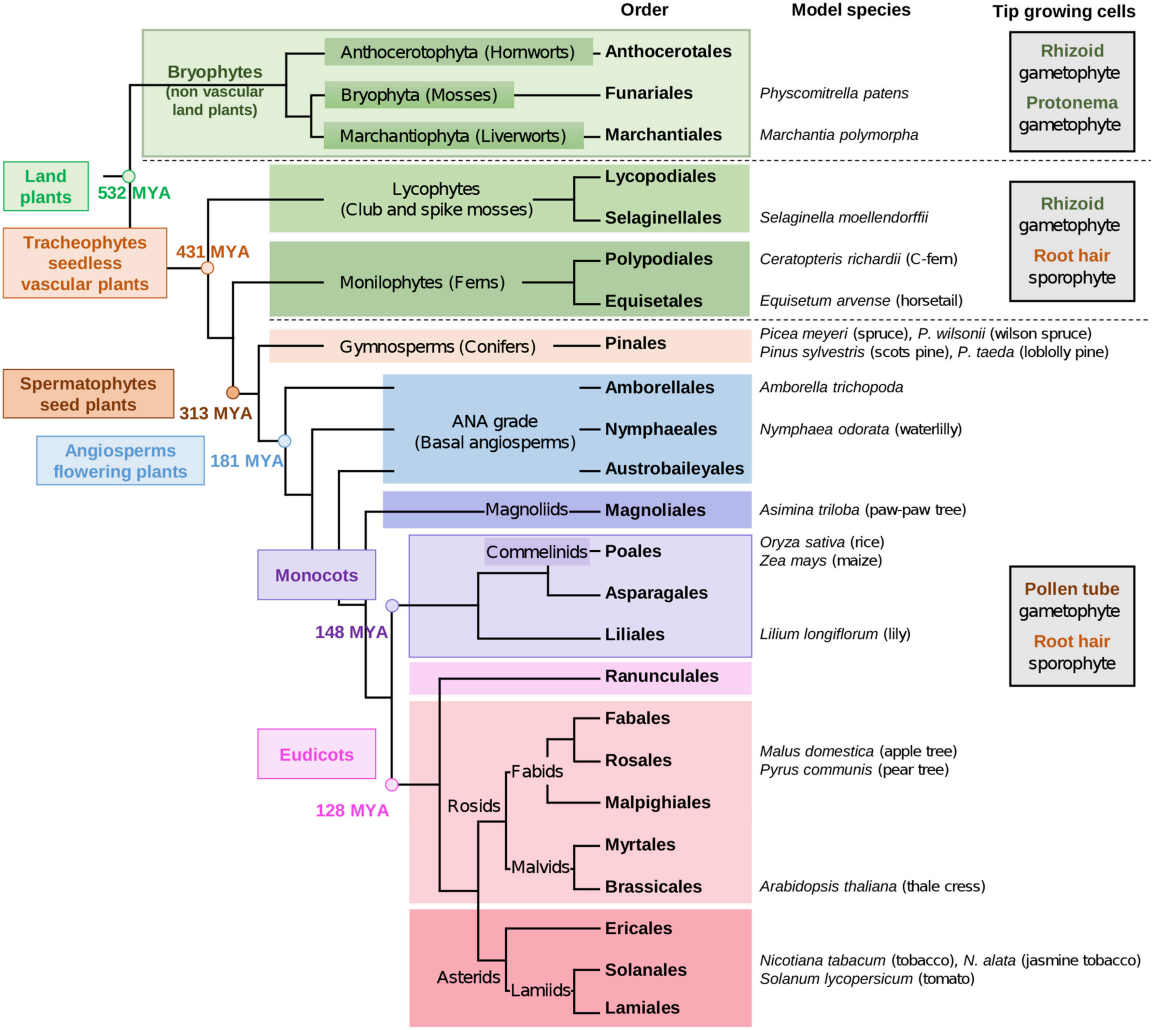
Phylogenetic tree of land plant lineages showing the occurrence of gametophyte *vs* sporophyte tip-growing cells according to Jones and Dolan (2012) and Rounds et al. (2011). The main studied species are indicated in the column “model species.” The phylogeny of land plants is according to Puttick et al. (2018) and The Angiosperm Phylogeny Group et al. (2016). The timescale was estimated by Kumar et al. (2017) and is indicated by millions of years ago (MYA).

The early non-vascular plants that successfully colonized land are thought to have been similar to extant bryophytes. Today, bryophytes include liverworts, mosses and hornworts. Later on, the seedless vascular plants appeared (lycophytes and monilophytes) (Figure 1). Bryophytes are characterized by a gametophyte stage of the life cycle. Then, during evolution, the gametophyte life cycle was reduced gradually, and was restricted solely to reproduction in seed plants (Niklas and Kutschera, 2010; Lora et al., 2016; Hackenberg and Twell, 2019). To date, more than 90% of land plants are the sporophyte-dominant flowering plants (Williams and Mazer, 2016).

The most studied seedless plant so far is the model moss *Physcomitrella patens* (Rensing et al., 2008). But recently, attention has turned to other species, such as the liverwort *Marchantia polymorpha* (Bowman et al., 2017), the lycophyte *Selaginella moellendorffii* (spike moss) (Figure 1) (Banks et al., 2011) and the monilophyte *Ceratopteris richardii* (C-fern) (Banks, 1999; Leroux et al., 2013) (Figure 1), for which the genome sequencing is under way. The genome sequencing of other species having gametophyte tip-growth will allow comparative genomics for ortholog genes to those of model seed plants, such as the monocot crop *Oryza sativa* (rice) (International Rice Genome Sequencing Project, 2005) and the eudicot *Arabidopsis thaliana* (The Arabidopsis Genome Initiative, 2000).

In the moss *P. patens*, gametophytic spores germinate and produce a multi-branched structure of filamentous protonemata. This structure consists of tip-growing chloronemal and caulonemal cells that will produce leafy gametophores anchored to the soil by another tip-growing structure called rhizoid (Menand et al., 2007). In addition to anchorage, rhizoids are of main importance for water and nutrient absorptions (Jones and Dolan, 2012). Atomic force microscopy and scanning electron microscopy have revealed that long fibrillar structures, disorganized in the caulonemal apical cells, become oriented in longitudinal arrays parallel to the growth axis in the proximal region (Wyatt et al., 2008) as it was observed in *A. thaliana* pollen tubes (Chebli et al., 2012). Menand et al. (2007) showed that the formation of rhizoids in *P. patens* is controlled by genes that are orthologs to those controlling the sporophyte root hair development in *A. thaliana*. In *M. polymorpha*, similar structures are also found and two types of rhizoids were described: rhizoids with small diameters and thick cell walls and smooth rhizoids with large diameters and thin cell walls (Cao et al., 2014), probably linked to their respective functions (anchorage, plant support or absorption). Honkanen et al. (2016) demonstrated also that several genes involved in rhizoid formation and growth in *M. polymorpha* were also involved in root hairs as Menand et al. (2007) mentioned on *P. patens* rhizoids. This reveals that the mechanisms for constructing the tip-growing cells with absorption and anchorage functions were conserved among land plants and were active in the earliest ones (Jones and Dolan, 2012). Without any doubt, all those tip-growing cells: rhizoids, protonemata, root hairs and pollen tubes share several common features (Crotty, 1967; Taylor et al., 1996). However, as suggested by Bascom et al. (2018), these structures must possess some differences as they are either short-lived (pollen tubes) or long-lived (protonemata, rhizoids) cells and they perform divergent functions.

In contrast with rhizoids and protonemata, which must sense external environmental signals, pollen tubes are specialized in carrying the sperm cells to the ovules and must sense the female environment cues allowing efficient guidance to the ovules and seed production (Higashiyama et al., 2003). To succeed in this process, the spatial and temporal controls of the pollen tube growth are critical within the female tissues: stigma, style and ovary. These organs vary greatly depending on the species: stigmas can be wet, semi-dry or dry; styles can be short, long, solid or hollow, ovary can contain a wide range of ovule numbers (Williams and Mazer, 2016). This will surely impact the duration and efficiency of reproduction.

Another interesting difference between those tip-growing cells is the growth rate. First, it has been shown in *P. patens* that caulonemal cells expanded faster (≈ 20 μm/h) than chloronemal cells (≈ 6 μm/h) (Menand et al., 2007). Secondly, an interesting survey presented by Williams et al. (2016) revealed that pollen tubes from the gymnosperms cycads/*Ginkgo* were the slowest growing cells with a growth rate between 1 and 5 μm/h. It is noteworthy that in these plants, pollen tubes grow like a haustorium rather than tip-growing cells. In conifers/gnetophytes, pollen tubes represented a major evolutionary step in the male gametophyte development of gymnosperms (Fernando, 2005) with a faster expanding pollen tube tip (1-15 μm/h) (Williams et al., 2016; Hackenberg and Twell, 2019). Gametophytic protonemata from *P patens* and rhizoids of mosses, liverworts and C-fern have growth rates ranging between 5–20 μm/h and 10–400 μm/h, respectively (Williams et al., 2016). The fastest tip-growing cells are angiosperms pollen tubes ranging from 10 to 20,000 μm/h with an average growth rate between 500–1,000 μm/h for most of the 180 species studied (Williams, 2012; Williams et al., 2016). Growth rate of pollen tubes has been obtained so far with *in vitro* experiments that consequently prevent the likely control of the tip-growth expansion by the female sporophyte (Lord, 2000). This wide difference of growth rates has an evident impact on the timing interval between pollination and fertilization which ranges from 10 h to about 12 months in gymnosperms and from 15 min to about 12 months in angiosperms (Williams, 2008). Fast-germinating pollen grains, fast-growing pollen tubes, pollen competition and performance, and the diversity of pollen tube pathways are major evolutionary traits. These characteristics linked to the fertilization rate are likely to be one of the reasons for the great success of angiosperms among the land plants (Lora et al., 2016; Williams et al., 2016; Williams and Reese, 2019).

One of the most important and common feature of those tip-growing cells is the need for the cell wall to be sufficiently elastic to allow cell expansion but stiff enough to resist against internal turgor pressure (Winship et al., 2010; Cameron and Geitmann, 2018). As a consequence, this type of growth requires a different cell wall organization compared to anisotropic growth. During tip-cell expansion, cell wall deposition and remodeling are required on a different time scale depending on the growth rate, to fine-tune the cell wall mechanical properties (Parre and Geitmann, 2005a,b; Roberts et al., 2012; Mollet et al., 2013; Rounds and Bezanilla, 2013; Vogler et al., 2013; Braybrook and Jnsson, 2016; Cameron and Geitmann, 2018). Most of the cell wall polymers are synthesized and/or processed in the Golgi apparatus, and secreted at the tip into the cell wall via Golgi-derived vesicles (Farrokhi et al., 2006) which fuse with the plasma membrane to sustain growth. These polymers include hemicelluloses: xylan and their derivatives arabinoxylan (AX), glucuronoarabinoxylans (GAXs), mannans, xyloglucans (XyGs); the pectin motifs: homogalacturonan (HG), rhamnogalacturonan type-I (RG-I) and type-II (RG-II) and the hydroxyproline-rich glycoproteins (HRGPs): arabinogalactan proteins (AGP) and extensins (EXTs). On the other hand, other cell wall polymers (cellulose and callose) are synthesized at the plasma membrane via CELLULOSE SYNTHASES (CESA) or CALLOSE SYNTHASES (CalS)/GLUCAN SYNTHASE-LIKE (GSL) complexes (Farrokhi et al., 2006).

One of the special features of most angiosperm pollen tubes is the presence at the tip of one cell wall layer (Roy et al., 1997) composed mostly of pectins, hemicellulose and little cellulose. In the shank of the tubes however, two layers are clearly visible with the inner layer mostly composed of callose and cellulose (Lennon and Lord, 2000; Dardelle et al., 2010; Chebli et al., 2012; Lampugnani et al., 2013). It is noteworthy that the inner cell wall layer is generally thinner in *in vivo*-grown pollen tubes than *in vitro* due to mechanical support of the female tissues within the transmitting tract of the style (Lennon et al., 1998; Lennon and Lord, 2000). In contrast, in the gymnosperms studied so far, only one cell wall layer has been observed in the shank of the pollen tube (Derksen et al., 1999; Yatomi et al., 2002; Fernando et al., 2010; Abercrombie et al., 2011). During evolution, cell wall components have appeared and evolved in composition and structure along with remodeling enzymes. Cellulose is present in many lineages (including animals), AGP motifs appeared in the Rhodophyta and persist in the entire green lineages, XyG and most pectins arose in the green lineages (starting in Charophyta), and β-(1,3),(1,4)-glucan (Mixed Linkage Glucans, MLG) were detected in Rhodophyta, Chlorophyta, monilophytes and in the monocot Poales (Figure 1) (Popper, 2008; Popper et al., 2011; Sørensen et al., 2011; Fangel et al., 2012) even though they seem to be absent in tip-growing cells.

This review highlights our current knowledge on the composition, distribution, and functional aspects of biosynthesis and remodeling of the main cell wall polymers including pectins, hemicelluloses, callose, cellulose, arabinogalactan-proteins and extensins during the tip-expansion of gametophyte cells (protonemata, rhizoids and pollen tubes) in bryophytes, pteridophytes (lycophytes and monilophytes), gymnosperms and the monocot and eudicot angiosperms.

## 2. Cellulose

### 2.1. Structure and Biosynthesis

Cellulose is one of the most common polymers found in living organisms. It is found in cyanobacteria as within the eukaryotic lineages, indicating an ancient origin (Nobles et al., 2001; Popper et al., 2011). Cellulose is a polysaccharide composed of β-D-glucopyranose (Glc) linked by (1–4) glycosidic bonds. In all land plants and some green algae, cellulose microfibrils are synthesized by a complex of 18 cellulose synthases A (CESA), according to computational analyses, instead of the 36 originally proposed, forming a rosette in the plasma membrane (McNamara et al., 2015; Oehme et al., 2015; Nixon et al., 2016; Jarvis, 2018). The resulting cellulose microfibrils are composed of probably 18 glucan chains (Vandavasi et al., 2016) with crystalline and desordered form of cellulose co-existing in each cross-section of the microfibril (Jarvis, 2018). Phylogenetic analyses have shown that *P. patens* and *S. moellendorffii* CESA protein sequences constitute their own clade while in the seed plants the phylogenetic tree is separated into 6 major clades indicating ancient gene duplication events (Carroll and Specht, 2011). The CESA6 clade is divided into 2 subclades: the first one (6A) is found only in eudicots while the other one (6B) is present in both monocots and eudicots. Arabidopsis seemed to have lost the 6B clade (Carroll and Specht, 2011).

The moss *P. patens* genome contains seven *CESA* genes named *PpCESA3-8* and *10* and three *CESA* pseudo-genes (*PpCESA1-2*, and *9*) (Roberts and Bushoven, 2007; Yin et al., 2009; Wise et al., 2011). Moreover, *Funaria hygrometrica* (Reiss et al., 1984; Rudolph and Schnepf, 1988; Rudolph et al., 1989) and *P. patens* (Roberts et al., 2012; Nixon et al., 2016) have rosette-type CES complexes. This reveals that cellulose synthesis in non-vascular plants is similar to Charophycean green algae and land plants than other algae which, depending on the species, have linear or other types of complexes (Tsekos, 1999; Saxena and Brown, 2005). Among the seven *CESA* expressed in *P. patens* protonemal cells, several may be involved in tip-growth (Tran and Roberts, 2016) and others are involved in the secondary cell wall biosynthesis of sclereid cells in the leaf midribs of *P. patens* (Norris et al., 2017). In ferns, analyses of genomes and transcriptomes have shown that *C. richardii, Pteridium aquilinum* and *Adiantum capillus-veneris* possess ortholog genes that clustered with *AtCESA1-4* and *AtCESA7-8* (Yin et al., 2014), but tissue specific expression has not been carried out, yet. In the genome of *A. thaliana*, ten *CESA* genes and six *CELLULOSE SYNTHASE LIKE D* (*CSLD*) are found (Richmond and Somerville, 2001; Bernal et al., 2008), while the rice genome contains seven *CESA* and five *CSLD* (Wang et al., 2010). In *A. thaliana*, CESA6, CSLD1 and CSLD4 were detected at the plasma membrane of the whole pollen tube (Wang et al., 2011; Chebli et al., 2012). *CESA1, 3*, and *9* are also expressed suggesting a possible role during pollen tube growth (Persson et al., 2007; Chebli et al., 2012). Similarly, in *Nicotiana tabacum* pollen tubes, CESA and CSLD were localized at the plasma membrane of the entire pollen tube with the highest level at the tip, driven by microtubules (Cai et al., 2011). To date, no information on the expression of CESA genes in pollen tubes of gymnosperms has been reported.

### 2.2. Localization in Tip-Growing Cells

Even if callose is the main component of the cell wall in angiosperm pollen tubes, cellulose, often detected with calcofluor white (which does not discriminate between cellulose and callose) (Supplemental Table 1), is generally located all along the pollen tube with a weaker detection at the tip than in the shank. This pattern was observed in *Pyrus communis* (Aloisi et al., 2017), *N. tabacum* (Ferguson et al., 1998), lily (Roy et al., 1997) and *A. thaliana* (Lennon and Lord, 2000; Derksen et al., 2002) (Figure 1). However, using CBM3a, a specific probe for cellulose (Supplemental Table 1), two populations of *A. thaliana* pollen tubes were observed: 70% were strongly labeled in the entire tube and 30% did not display any labeling at the tip (Chebli et al., 2012), probably depending on the growth status and/or the developmental stages of the pollen tube. At the sub-cellular level, cellulose was located in the inner cell wall layer of the pollen tube and in vesicles (Chebli et al., 2012). This suggests that cellulose biosynthesis might be initiated in vesicles carrying CES that will be deposited at the tip. Alternatively, these vesicles could originate from endocytosis (Chebli et al., 2012) suggesting cellulose recycling.

In gymnosperm pollen tubes, it has been reported that cellulose, stained with calcofluor white, is present all along the pollen tube in *Picea abies* (Lazzaro et al., 2003) and *Picea wilsonii* (Sheng et al., 2006). However, it is noteworthy that calcofluor labels β-glucans, and does not discriminate cellulose from callose (Supplemental Table 1). In *Pinus sylvestris* (Figure 1), microfibrils appear to be less dense in the pollen tube tip compared to the rest of the pollen tube wall as shown from alkaline or acid-treated pollen tubes observed by transmission electron microscopy (Derksen et al., 1999).

In mosses, cellulose is present in the cell wall of all species that have been investigated, including *P. patens* protonemal cells (Kremer et al., 2004; Moller et al., 2007; Nothnagel and Nothnagel, 2007; Goss et al., 2012; Roberts et al., 2012). In contrast, *P. patens* rhizoids were very weakly labeled with CBM3a (Berry et al., 2016). Cellulose probed with CBM28 (Supplemental Table 1) was not detected in the protonemal cells or rhizoids (Berry et al., 2016). However, it was shown that those two probes recognize different types of cellulose, display different binding affinity and that other cell wall polymers such as pectin can reduce their interactions with cellulose (Blake et al., 2006). In ferns, such as *C. richardii*, calcofluor white staining showed a uniform labeling on the entire rhizoid cell wall, suggesting the presence of β-glucan, cellulose or callose, or both all along the cell (Eeckhout et al., 2014). Finally, in the cell wall of *C. richardii* antheridia, CBM3a staining confirmed also the presence of cellulose (Lopez and Renzaglia, 2018). Altogether, the data demonstrate that cellulose is broadly present in all these tip-growing cells at different levels.

### 2.3. Role in Tip-Growth

In fast-growing pollen tubes, despite its low abundance compared to sporophytic cell wall tissues, studies using *A. thaliana* mutants have shown that cellulose/hemicellulose synthesis is of main importance. AtCSLD1 and AtCSLD4 are two membrane-localized proteins in pollen tubes. *Csld1* and *csld4* homozygous mutants are sterile, and mutant pollen grains showed abnormal germination with high rates of ruptured tubes *in vitro*, and arrest of growth *in vivo* (Wang et al., 2011). Mutation in *AtCSLC6* resulted in a strong reduction of pollen tube growth (Boavida et al., 2009). However, it has been reported that CSLC5 was involved in XyG backbone synthesis (Liepman et al., 2010). Similar results were observed in the tip-growing rhizoids of *M. polymorpha* (Honkanen et al., 2016). Generation of mutant lines in *M. polymorpha* showed that 33 genes were required for rhizoid growth (Honkanen et al., 2016). Among them, mutations in *MpCSLD1* and *MpCSLD2* which have been suggested to function in synthesis of mannan, xylan and cellulose (Gu et al., 2016), induce very short and burst tips or short rhizoids, respectively (Honkanen et al., 2016), supporting the importance of the cellulose-hemicellulose network in the mechanical strengthening of the cell wall.

Cellulose synthesis inhibitors 2,6-dichlorobenzonitrile (DCB) and isoxaben strongly affect the growth of tip-growing cells, particularly pollen tubes. In pollen tubes of *Lilium auratum, Petunia hybrida* (Anderson et al., 2002) and *Pinus bungeana* (Hao et al., 2013), DCB induced distortion of the cell wall, changes in the cell wall composition and tip-bursting (Anderson et al., 2002). Isoxaben, induced tip swelling in conifer pollen tubes (Lazzaro et al., 2003). Interestingly, in *P. patens*, DCB and isoxaben, treatments did not have a strong effect on protonemal growth rates, and only DCB caused tip rupture (Tran et al., 2018). Those treatments seemed to modify the CESA mobility but not the density and probably have an impact in the patterning of cellulose biosynthesis in protonemal cells (Tran et al., 2018). These data reveal a clear difference between pollen tubes and protonemal cells in response to those cellulose synthesis inhibitors, probably due to the different levels of cellulose found in those cells. However, moderate enzymatic treatment with cellulase resulted in larger pollen tube diameters, promoted tip swelling and eventually bursting (Aouar et al., 2010). All these data support that despite the low abundance of cellulose in the cell wall of pollen tubes, it has an important function together with hemicelluloses in maintaining the cell wall integrity (Mollet et al., 2013; Tran et al., 2018).

Because of their crystalline nature, the formation of cellulose microfibrils is generally associated with the stiffening and the reduction of extensibility of the cell wall. In most cells with cylindrical geometry, cellulose microfibrils are oriented perpendicular to the growth axis (Baskin, 2005) in order to strengthen the cell wall against turgor-induced tensile stress in a circumferential direction and to ensure elongation in the longitudinal direction. In tip-growing cells, cellulose microfibrils are organized differently. In pollen tubes from *Petunia* and *Pinus*, the principal orientation of cellulose microfibrils is at 45° to the long axis (Sassen, 1964; Derksen et al., 1999). It is observed in *Lilium* and *Solanum* pollen tubes an orientation at 20° and 15°, respectively (Aouar et al., 2010; Geitmann, 2010). In regards to this pattern of deposition, it is admitted that cellulose does not play an important role in resisting circumferential tensile stress in the distal tubular region of the pollen tube and in tip-growing cell in general. But the fact that inhibitors of cellulose synthesis cause apical swelling in pollen tubes indicates that cellulose microfibrils affect cell wall stability in the transition region between the tip and the sub-apical dome.

## 3. Hemicelluloses

Hemicelluloses are a large family of cell wall polysaccharides including xylans, mannans, (1-3)(1-4)-β-glucans (mixed-linkage glucans) (MLG) and xyloglucans (XyGs) (Scheller and Ulvskov, 2010). MLG is narrowly found in the plant kingdom. This polymer has been described in Poales (transiently found in young and growing tissues), horsetails and several algae (Fry et al., 2008; Sørensen et al., 2008; Salmeán et al., 2017). However, a recent study showed that it could also be found in other commelinid and non-commelinid monocot plants suggesting a larger distribution across plant lineage as previously thought (Figure 1) (Little et al., 2018). Using BS-400-3 (Supplemental Table 1), MLG was not detected in *P. patens* protonemata (Moller et al., 2007) and no information is available so far on its presence in pollen tube cell walls. Thus, this polymer, despite its possible important function, will not be further discussed.

### 3.1. Polymers

#### 3.1.1. Xylans

**Figure 2:**
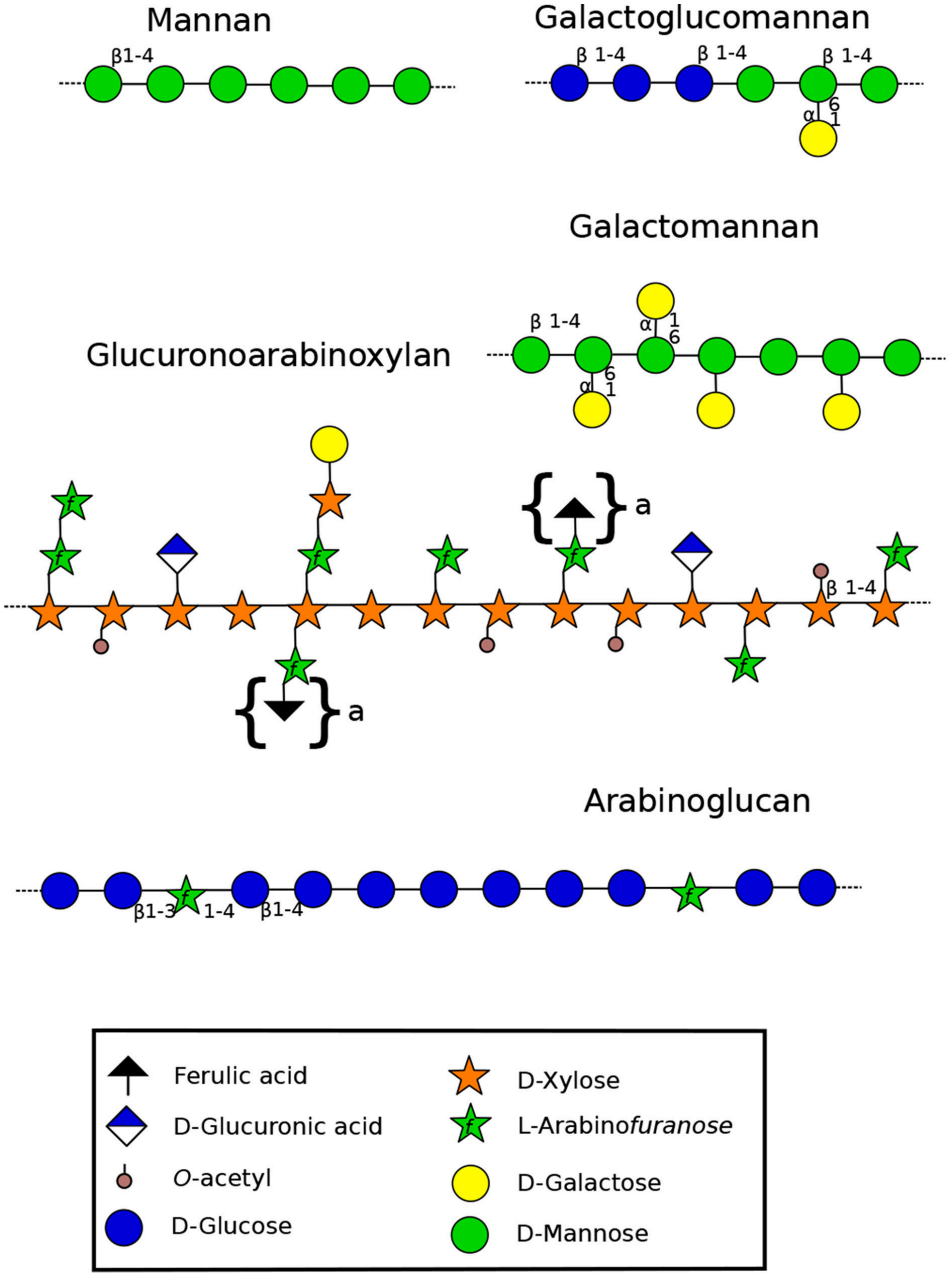
Structure of hemicelluloses found in the cell wall of land plants (Scheller and Ulvskov, 2010). Xyloglucans are represented in Figure 3. Mannan-type hemicellulose can be found as mannans, galactomannans and galactoglucomannans. Xylan-type hemicellulose has been described as xylans, arabinoxylans (AX) and as represented in the figure: glucuronoarabinoxylans (GAXs). Arabinoglucans were recently described in the moss *P. patens* (Roberts et al., 2018). Structures under braces are only found in monocots. β-(1-4)(1-3)-mixed linkage glucans, not discussed in this review, are not represented. Monosaccharides are represented according to the Symbol Nomenclature for Glycans (SNFG) (Varki et al., 2015), at the exception of the ferulic acid which is not present in the SNFG. Ferulic acid is represented with a black triangle.

Xylans (Figure 2) consist of a β-(1-4)-linked D-xylose (Xyl) backbone that can be substituted by L-arabinose (Ara), D-galactose (Gal), glucuronic acid (GlcA) and acetyl groups (Scheller and Ulvskov, 2010). Even though *A. thaliana* root hairs are labeled with LM10 and LM11 (Supplemental Table 1) (Larson et al., 2014), it seems that xylans and AX are not present in detectable levels in pollen tubes, as 4-Xyl was not detected (Dardelle et al., 2010). However, 4-Xyl was detected in very low level (<1%) in *N. alata* pollen tubes (Lampugnani et al., 2013). To date, no information is available on the presence of this polymer in monocot nor in gymnosperm pollen tubes. However, reports have shown that xylanases, present in the pollen coat (sporophytic origin), were important to facilitate the pollen tube penetration into the silk female tissues in maize (Suen and Huang, 2007). Kulkarni et al. (2012) confirmed the presence of xylans in the cell wall of leafy tissues and axillary hair cells of *P. patens* but a lack of LM10 and/or LM11 binding was observed in the liverwort and moss rhizoids and protonemata (Carafa et al., 2005; Eeckhout et al., 2014; Berry et al., 2016). In addition, AX was not detected in *P. patens* by comprehensive microarray polymer profiling (CoMPP). However, linkage analysis has revealed the presence of 4-Xyl (Moller et al., 2007) suggesting its presence but in low level. In contrast, leafy-tissues of *Selaginella* sp. have a cell wall enriched in *O*-acetylated xylan, AX and GAX (Plancot et al., 2019), but no information is available so far on rhizoids. This may suggests that xylans are not of main importance for tip-growing cells of the species studied so far.

#### 3.1.2. Mannans

Mannans are ancient polymers found in many green lineages (Voiniciuc et al., 2018). Its content started to decrease in eudicots, and low levels are found in the secondary cell wall of eudicots except in seeds where the amount can be high (Rodríguez-Gacio et al., 2012). Several structures have been characterized including mannans, galactomannans, glucomannans and galactoglucomannans (Figure 2). Each of these polysaccharides contains a backbone of mannose (Man) linked in β-(1-4) or a combination of Glc and Man that can be substituted by α(1-6)-linked Gal (Figure 2) (Moreira and Filho, 2008). Members of *CSLA* family are present in diverse land plant species, and several of them encode mannan synthases responsible of the synthesis of β-1,4-mannan backbone of galactomannans and glucomannans *in vitro* and probably *in vivo* (Liepman et al., 2005; Rodríguez-Gacio et al., 2012;Kaur et al., 2017).

Glycan microarray analysis revealed that mannans and glucomannans, probed with BS-400-4 (Supplemental Table 1), were abundant in *P. patens* protonemata and that 4-Man and 4,6-Man represented about 7% of the total cell wall (Moller et al., 2007). Using the same probe, these polymers were labeled in the whole protonema (caulonemal and chloronemal cells) with a strong labeling intensity in the transverse cell wall of the filament, and rhizoids (Liepman et al., 2007; Berry et al., 2016). In *A. thaliana*, glycan microarray analysis showed that mannans are distributed in the whole plant and are especially abundant in flowers, siliques, and stems (Liepman et al., 2007). However, linkage analyses of *N. alata* and *A. thaliana* pollen tubes did not reveal the presence of 4-Man suggesting that if present, mannans are a minor component in those tip-growing cells. Since mannans are found in rhizoids and protonemata, but have not been detected in pollen tubes so far, it seems to indicate that they might be of importance for *P. patens* tip-growth, unlike in pollen tubes. Specialization of tip-growing cells from the multi-task protonemata and rhizoids, to pollen tubes devoted to carry the sperm cells, would indicate that mannans were required for other functions (anchorage, nutrient uptake/water sensing, interaction with soil particles and/or micro-organisms, cell wall reinforcement…) that pollen tubes lost over specialization. Interestingly, mannans can cross-link cellulose microfibrils as do other hemicelluloses like xylans and XyGs (Scheller and Ulvskov, 2010).

The coexistence of mannans and cellulose in *P. patens* may reinforce the protonemal and rhizoid cell walls that are subjected to hydrated/de-hydrated cycles. The recent paper by Plancot et al. (2019) on desiccation tolerant *Selaginella* sp. which have a cell wall enriched in *O*-acetylated mannan, and studies on resurrection plants (Moore et al., 2009), have suggested that this ancient polymer could also serve as a cell wall desiccation protectant. In pollen tubes, due to their growth in the female transmitting tract, this polymer was un-necessary and callose may have replaced mannans for this role.

#### 3.1.3. Xyloglucans

**Figure 3:**
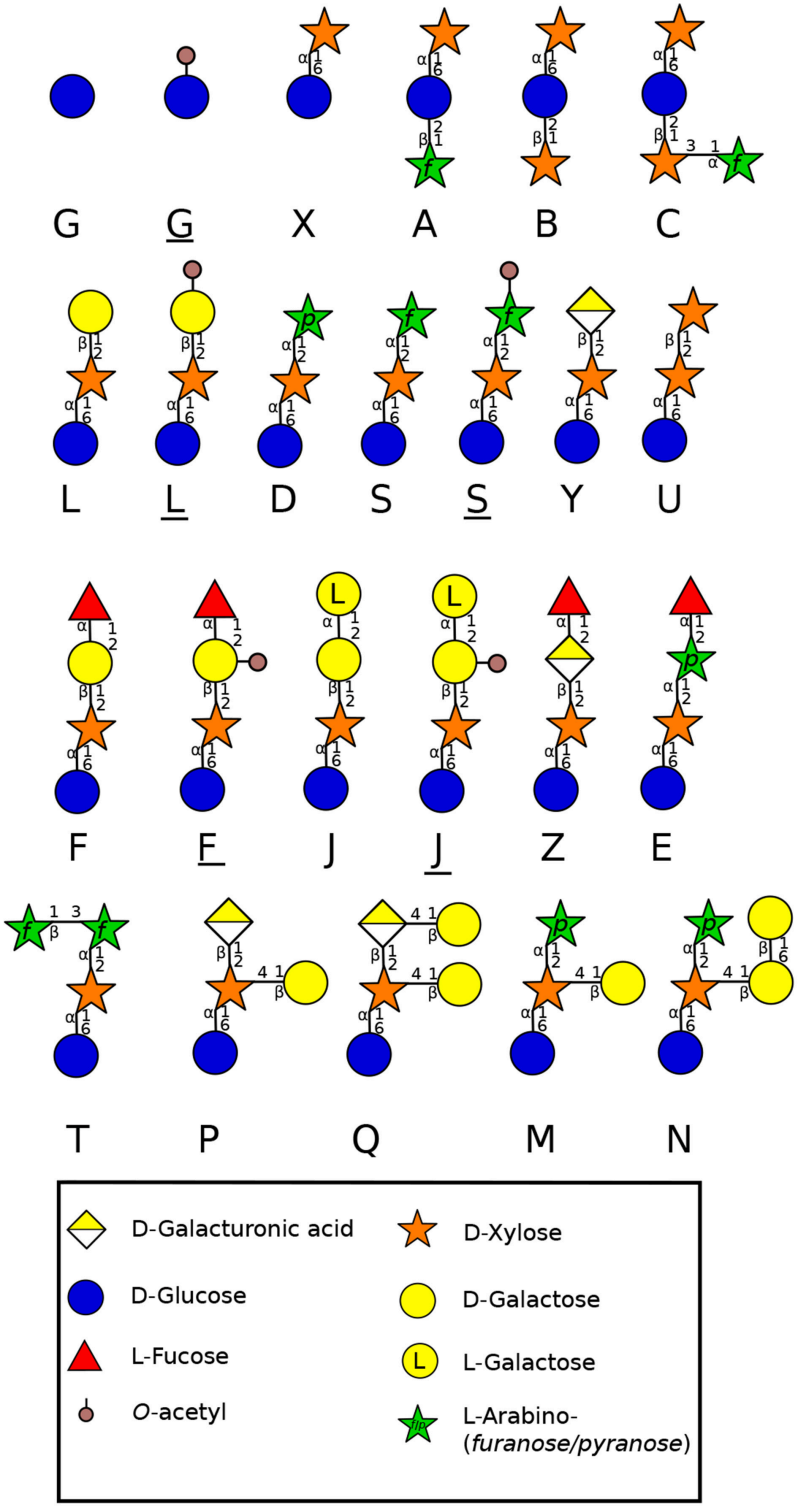
Side chain diversity found in XyG throughout land plant lineages according to Schultink et al. (2014) using the one letter code proposed by Fry et al. (1993). Underlined letters correspond to *O*-acetylated side chains. Monosaccharides are represented according to the Symbol Nomenclature for Glycans (SNFG) (Varki et al., 2015).

XyG is the most abundant hemicellulosic polysaccharide in the primary cell wall of eudicots and non-commelinid monocots (O'Neill and York, 2003). Micro-domains of XyG interact directly with cellulose microfibrils by non-covalent bonds (Park and Cosgrove, 2015) and participate in the regulation of the cell wall strengthening during cell growth (Scheller and Ulvskov, 2010). In eudicots, XyG is a XXXG-type. This type of hemicellulose is composed of a β-1-4 glucan backbone which can be substituted by different side chains (Figure 3) named by the one letter code nomenclature proposed by Fry et al. (1993). In most eudicots, the Xyl residue (X side chain) can be branched on the C-2 with a Gal (L side chain), itself substituted by fucose (Fuc) (F side chain) forming a fucogalactoXyG (Schultink et al., 2014). Surprisingly, in several other eudicots including the lamiid clade and particularly the Solanales (Figure 1) (The Angiosperm Phylogeny Group et al., 2016), XXGG and XXGGG-types are found. In these plants, the Xyl residue is not substituted with Gal-Fuc but instead with Ara (S side chain) or Ara-Ara (T side chain) (Hoffman et al., 2005; Schultink et al., 2014) forming an arabinoXyG. Several of these sugars can be *O*-acetylated (Glc, Ara or Gal) to prevent enzymatic degradation (Schultink et al., 2014). Interestingly, in contrast with the sporophytic cells, XyG in pollen tubes of the Solanales (as shown in *N. alata, N. tabacum* and several tomato species) contains L and F side chains (Lampugnani et al., 2013; Dardelle et al., 2015), suggesting that F motifs may be an important structural feature of fast-growing/short-lived pollen tubes.

The moss *P. patens* protonema contains a XXGGGG-type decorated with X and L side chains found in angiosperms, but also other motifs containing GalA (P and Q side chains) and branched Xyl residues (M and N side chains) (Figure 3) (Peña et al., 2008). The aerial gametophyte of the liverwort *M. polymorpha* produces a XXGG-type with X, L, P and Q motifs (Figure 3) (Schultink et al., 2014). The functional aspect of these differences in composition are not clear, but the importance of GalA containing side chains was highlighted in the tip-growing rhizoids of *M. polymorpha*. Mutation in a XyG-specific galacturonosyltransferase (*MpXUT1*) gene induced very short rhizoids that ultimately burst at the tip (Honkanen et al., 2016). *A. thaliana* root hairs contain also XyG with Xyl-GalA (Y side chain) and Xyl-GalA-Fuc (Z side chain) (Figure 3) (Peña et al., 2012), which are not found in the cell wall of other parts of the plant, revealing the importance of GalA and GalA-Fuc motifs in the cell wall strengthening and tip-growth of these anchorage and/or water absorbing cells.

The lack of fucosylated XyG in liverworts and mosses shows that fucosylation of XyG arose later, probably in hornworts. In several gametophyte hornworts including *Anthoceros agrestis* (Figure 1), XyG is of the XXXG-type harboring low levels of fucosylated motifs, a common structure found in most eudicot plants (Peña et al., 2008; Hsieh and Harris, 2012; Schultink et al., 2014). Rhizoids have also a different composition assessed by cell imaging. No or weak labeling was observed with LM15 (Supplemental Table 1) on rhizoids of *P. patens* (Berry et al., 2016) whereas rhizoids were strongly labeled with LM15 in the monilophyte *C. richardii* (Eeckhout et al., 2014). This is in agreement with the specificity of LM15 which recognizes XXXG and not the XXGGGG motifs.

#### 3.1.4. Arabinoglucans

*P. patens* produces a Glycosyl Transferase (GT) (*Pp3c124670*) similar to MLG synthases found in ascomycetes (Roberts et al., 2018). This protein appears to be involved in the synthesis of a new type of cell wall component: an unbranched glucan punctuated by Ara. This new arabinoglucan (Figure 2) has not been described before. However, some similar GT genes present in algae, bryophytes, lycophytes and monilophytes make it possible to consider that a similar polymer might exist in other species (Roberts et al., 2018). Even though arabinoglucan is quite similar in structure to MLG, it is unlikely that this *P. patens* GT has led to the synthesis of MLG in monocots due to the important differences with MLG synthases. Thus, the appearance of MLG in monocots (Poales) would be from an independant origin (Roberts et al., 2018).

## 4. Pectins

**Figure 4:**
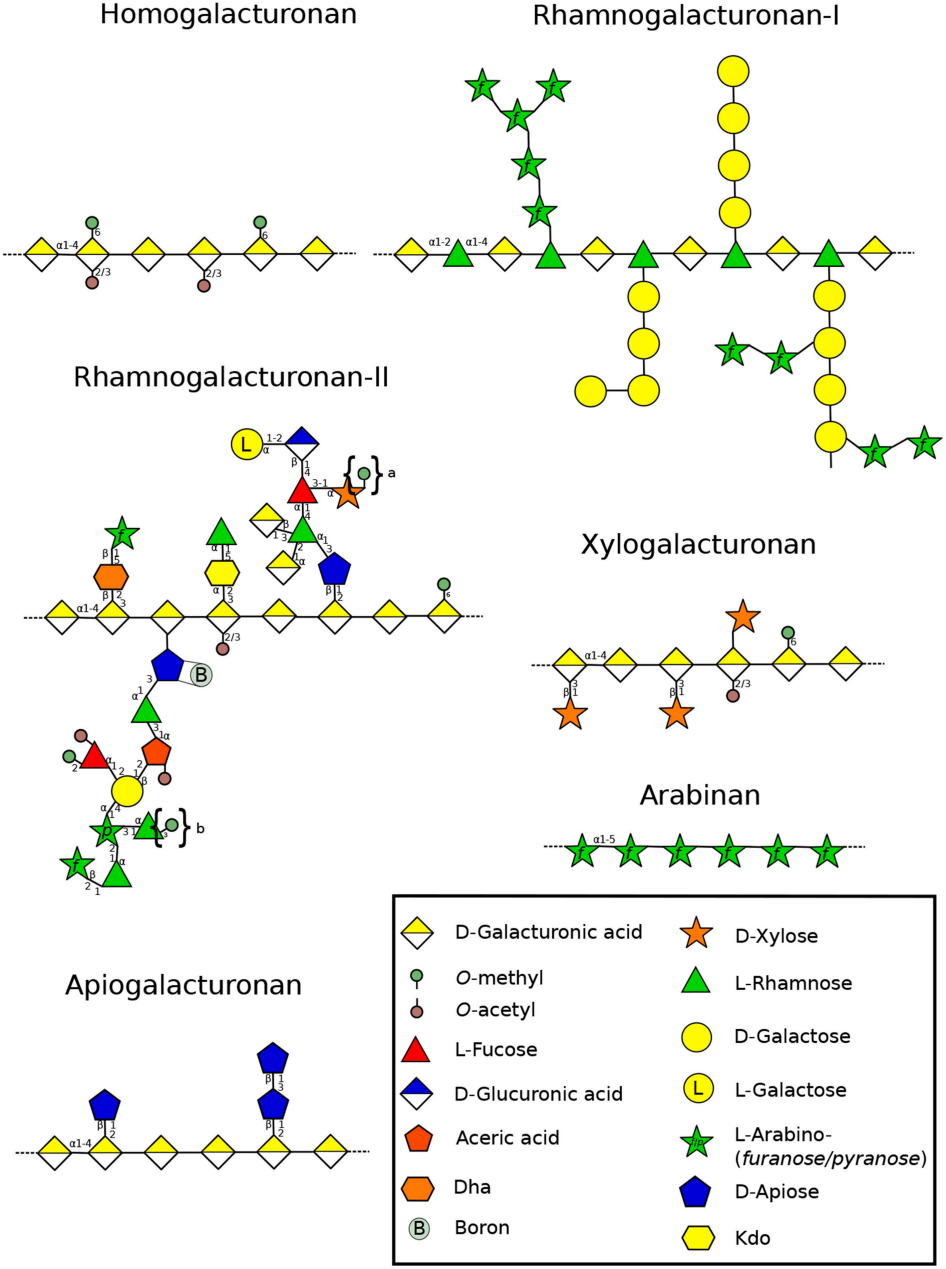
Structure of the different pectin domains found in the cell wall throughout land plant lineages (Wolf et al., 2012). The structural changes of RG-II side chains is presented according to Matsunaga et al. (2004), O'Neill et al. (2004), Pabst et al. (2013), and Ndeh et al. (2017). Linear arabinans were described in *N. alata* pollen tubes (Lampugnani et al., 2016). Symbols in braces represent methyl decorations: “a,” 0, 1, or 2 methylester(s) that can be found in Lemnoidae, and “b,” methylesters found only in bryophytes (Matsunaga et al., 2004; Avci et al., 2018). Dha: 3-Deoxy-D-lyxo-hept-2-ulopyranosaric acid, Kdo: 3-Deoxy-D-manno-oct-2-ulopyranosonic acid. Monosaccharides are represented according to the Symbol Nomenclature for Glycans (SNFG) (Varki et al., 2015).

Pectin is a major component of the primary cell wall in all organs of terrestrial plants (roots, leaves, stems and flowers) and is also present in tip-growing gametophytes, for which pectin plasticity or stiffness offers a variety of properties necessary for polarized growth. It represents about 33% of the primary cell wall (Atmodjo et al., 2013) except in the Poales in which pectin levels are lower (5–10%) (Smith and Harris, 1999). Pectins are generally described by four domains (Figure 4). Homogalacturonan (HG), a polymer made of (1,4)-GalA, that can be substituted with methyl or acetyl groups which will influence the remodeling and mechanical properties of the cell wall. Indeed, the high level of negative charges in weakly methylesterified HG (originating from the demethylesterification of GalA residues), makes HG an important domain for strengthening the cell wall by forming a complex between several HG chains and calcium constituting the so-called “egg-box” (Peaucelle et al., 2012). Removal of methyl and acetyl groups is coordinated by pectin methylesterases (PME) and pectin acetylesterases (PAE), whereas the cleavage of the HG backbone is controlled by polygalacturonases (PG) or pectate lyases (PL) (Pilnik and Rombouts, 1981). HG can also be substituted with Xyl or Apio residues forming xylogalacturonan (XylGalA)(Figure 4) and apiogalacturonan (ApioGalA) (Figure 4) (Avci et al., 2018). The latter is found abundantly in the fast-growing aquatic monocots such as eelgrass (*Zostera marina*), (Gloaguen et al., 2010) or lemnoids including *Lemna minor* (Avci et al., 2018). In the *Wolffiella* genus from the same Lemnoideae subfamily, ApioGalA decreases significantly and is replaced by XylGalA (Avci et al., 2018), suggesting that Apio was replaced by Xyl during evolution. Rhamnogalacturonan type I (RG-I) is composed of the repeating GalA-rhamnose (Rha) disaccharide that can be substituted on the Rha residues with various side chains, such as galactan, arabinan and type-I arabinogalactan (Atmodjo et al., 2013). Rhamnogalacturonan type-II (RG-II) has a HG backbone with 4-5 fairly well defined oligoside side chains containing unusual sugars such as Apio, aceric acid or Kdo (Dumont et al., 2016) (Figure 4). Moreover, RG-II forms a dimer *in muro via* borate cross-linking with two Apio (O'Neill et al., 2004). Mosses contain very low level of RG-II-like structure with methylated Rha (Matsunaga et al., 2004). Despite the fact that they are able to synthesize UDP-Apio and secondary metabolites containing Apio, they lack the GT machinery required for synthesizing Apio-containing cell wall polymers (Pičmanová and Møller, 2016; Smith et al., 2016). In contrast, in vascular plants, a comparable amount of borate-cross-linked RG-II is found and little composition changes were described across land plant lineages. These differences include different levels of methylation of Xyl in Lemnoidae, the presence of a methylated GlcA, L-Fuc or L-Gal in *A. thaliana* and other eudicot plants (Matsunaga et al., 2004; Pabst et al., 2013; Avci et al., 2018) (Figure 4).

### 4.1. Homogalacturonan

#### 4.1.1. Distribution and Composition

CoMPP analysis of *P. patens* revealed that highly methylesterified HG (Figure 4) probed with JIM7 (Supplemental Table 1) was only detected in a sporophyte-enriched sample and was very low in caulonemal and chloronemal-enriched fractions (Moller et al., 2007). In contrast, weakly methylesterifed HG was strongly detected with JIM5 in a gametophyte-enriched cell wall extract (Moller et al., 2007). As summarized in Table 1, concordant results were obtained by cell surface immuno-detection of the protonemal cells with JIM5 and LM19 showing a strong labeling in the whole protonemal filament (Lee et al., 2005; Berry et al., 2016). In contrast, in the rhizoid cell walls of *P. patens* and *C. richardii* a weak signal was found with LM19 on the entire rhizoid surface and no labeling was observed with LM20 (Eeckhout et al., 2014; Berry et al., 2016), suggesting that low levels of HGs are present in rhizoids or HG epitopes have been masked by other polymers.

**Table 1:** HG localization in expending tip-growing gametophytes (rhizoid, protonema, and pollen tube) grown *in vitro* across several plant lineages.

**Species**	**Probe**	**Cell type**	**Specimen treatment**	**Labeling pattern/effect**	**References**
**Bryophytes**					
*Physcomitrella patens*	JIM5, LM19	Protonema	Cell surface	- At the shank of protonema and in the tip	Lee et al., 2005; Berry et al., 2016
	LM19, LM20	Rhizoid		- No HGs detected on rhizoids	Berry et al., 2016
**Monilophytes**					
*Ceratopteris richardii*	LM19, LM20, LM18	Gametophyte	Alcohol insoluble residues	- Detection with LM18 and LM19	Eeckhout et al., 2014
		Rhizoid	Cell surface	- Only LM19 detected in the whole rhizoid	
				- No labeling with LM20	
**Gymnosperms**					
*Pinus sylvestris*	JIM5	Pollen tube	Cell surface	- At the shank, but weak	Derksen et al., 1999; Yatomi et al., 2002
	JIM7			- Strong at the tip and behind, ring-like in the old part of the pollen tube	
*Picea meyeri*	JIM5	Pollen tube	Cell surface	- Present at the shank of pollen tube	Chen et al., 2007
	JIM7			- Present at the tip only	
Angiosperms^*^					
**Monocot non-commelinid**					
*Lilium longiflorum*	JIM5	Pollen tube	Cell surface	- Entire surface of the pollen tube	Jauh and Lord, 1996
	JIM7			- Tube tip	
**Eudicots**					
**Brassicales**					
*Arabidopsis thaliana*	JIM5	Pollen tube	Cell surface	- Shank of the pollen tube only	Dardelle et al., 2010; Chebli et al., 2012
	JIM7			- Strong at the tip	
**Solanales**					
*Nicotiana tabacum*	JIM5	Pollen tube	Cell surface	- Strong in the shank of the pollen tube	Bosch et al., 2005
	JIM7			- Strong at the tip and weak in the shank	
**Lamiales**					
*Olea europaea*	JIM5	Pollen tube	Cell surface	- Uniform labeling in the whole pollen tube	Castro et al., 2013
	JIM7			- Strong at the tip, and weak in the shank	

* *For more descriptions of angiosperm pollen tubes, see Li et al. (1994)*.

In slow-growing gymnosperm pollen tubes, HGs were weakly labeled with JIM5 (Yatomi et al., 2002) (Table 1). Low methylesterified HGs are weakly detected in *P. sylvestris* pollen tubes and methylesterified HGs are found uniformly at the tip, in the young part of the tube and as a ring-like pattern in the oldest parts (Derksen et al., 1999) (Table 1). Those results suggest that de-methylesterification of HG occurs far back from the sub-apical dome in *P. sylvestris*. This was not the case in *P. wilsonii* where low methylesterified HGs were detected in the whole tube with a decline of intensity at the tip, while highly methylesterified HGs were present at the tip but extended way back from it (Chen et al., 2008). However, in an other species, *Picea meyeri*, Chen et al. (2007) have shown a strong labeling with JIM5 in the shank of the pollen tube and no JIM7 labeling was observed in this region but a strong labeling was detected at the very tip (Table 1). This kind of distribution (clear separation between highly and weakly methylesterified HGs) is generally observed in fast-growing angiosperm pollen tubes. *Pinus* species display generally a very low growth rate of their pollen tubes. For example, *Pinus strobus* pollen tubes grown *in vitro* were shown to expand at 0.59 μm/h (Williams, 2012) whereas *P. meyeri* pollen tubes grow at 13.2 μm/h (Chen et al., 2007), which is in the range of the fastest *in vitro*-grown gymnosperm pollen tubes (Williams, 2012). This 22-fold increases in growth rate may explain this structural shift from low to high levels of weakly methylesterified HG in the shank of the pollen tube cell wall. Indeed, similar labeling patterns were found in fast-growing angiosperm pollen tubes (Table 1) (Jauh and Lord, 1996; Bosch et al., 2005; Dardelle et al., 2010; Chebli et al., 2012; Castro et al., 2013). The monocot *Lilium longiflorum*, the eudicots *A. thaliana* and *N. tabacum* display an *in vitro* growth rate of 350–550 μm/h (Van-Hemelryck et al., 2017), 25–100 μm/h (Boavida and McCormick, 2007; Dardelle et al., 2010; Chebli et al., 2012) and 240–450 μm/h (Parton et al., 2003), respectively.

The labeling for weakly methylesterified HG in the shank is very often observed as ring-like pattern due to the oscillatory growth. The presence of highly methylesterified HG in the tip region allows sufficient elasticity to promote pollen tube growth. This level of methylesterification gradually decreases of two-third in the 10-12 μm from the tip (Chebli et al., 2012). In this region, a 4-fold increase of low methylesterified HG appears and remains stable in the shank that will gradually (between 2 and 12 μm from the tip) strengthen the mechanical property (increase of stiffness) of the cell wall with calcium (Chebli et al., 2012).

#### 4.1.2. Biosynthesis

HG is a well conserved cell wall component implying highly conserved biosynthetic genes. The GAUT superfamily (galacturonosyltransferase), member of the CAZy (Carbohydrate-Active enZYmes) GT8, is responsible for the biosynthesis of HG (Sterling et al., 2006; Wang et al., 2013). *A. thaliana* genome contains 15 *GAUT*s, and 10 *GAUT*-related genes *GATL* (*GAUT*-like) with functions in HG biosynthesis that remain unclear (Kong et al., 2011). The 15 *AtGAUT*s are in three well resolved clades and one unresolved polytomy (McCarthy et al., 2014). The 8 *PpGAUT* from *P. patens* are in only two of these clades and the polytomy (McCarthy et al., 2014). Among the 15 *AtGAUT*, only two (*AtGAUT13* and *AtGAUT14*) are predicted to be expressed in pollen with two GATL (*AtGATL4* and *AtGATL7*) (Qin et al., 2009; Kong et al., 2011; Mollet et al., 2013). One study on *A. thaliana* has shown that *AtGAUT13* and *AtGAUT14* have a redundant function, as a single mutation in one of the two genes did not show any phenotype (Wang et al., 2013). In contrast, the *gaut13/gaut14* double mutant was severely impaired in growth with swollen pollen tubes and abnormal cell wall organization (Wang et al., 2013), revealing the importance of HG in the cohesion of the cell wall at the tip and in the shank of the pollen tube. *GAUT13* and *GAUT14* are in the same clade than 3 *PpGAUT* from *P. patens* and 1 *SmGAUT* from *S. moellendorffi*. Thus, it is tempting to speculate that those genes may be involved in the tip-growth of protonemata and/or rhizoids.

#### 4.1.3. Remodeling

The level of methylesterifcation of HG is regulated by pectin methylesterases (PMEs) that convert highly esterified HG to weakly esterified HG. Depending on the mode of action of PMEs, “block” or “random,” the impact on the cell wall mechanics will be different. By consecutive de-methyl-esterification of HG, the cell wall will be stiffer due to the formation of calcium-HG complexes. In contrast, random action of PMEs will favor cleavages by polygalacturonases (PGs), reducing the stiffness of the cell wall (Sénéchal et al., 2014).

PMEs belong to 2 classes, type-I (group 2) contains only two to three introns and a long pro-region, while type-II (group 1) PMEs contain five or six introns coding the catalytic domain and no pro-region (Micheli, 2001; Sénéchal et al., 2014). In the type-I (group 2) PMEs, the pro-region contains sequence similarities with PME inhibitors (PMEI). This pro-region domain is suspected to inhibit the PME activity during its traficking to the cell wall (Micheli, 2001; Sénéchal et al., 2014).

*Populus tricocarpa* and *A. thaliana* have 88 and 66 PME genes, which is much higher than in the monocot *O. sativa* (41 genes) (Pelloux et al., 2007; Sénéchal et al., 2014) but the HG content in their cell walls is low. Sequence analysis predicts that *P. patens* and *S. moellendorffi* genomes contain fewer PMEs (14 and 11, respectively) (McCarthy et al., 2014) but no specific information is available on how many of them are expressed in protonemata (Moller et al., 2007; Pelloux et al., 2007; McCarthy et al., 2014). Transcriptomic analyses have revealed that *A. thaliana* expresses 14 pollen specific PMEs (Leroux et al., 2015). Among them, three studies (VANGUARD1 and VANGUARD-homolog, AtPPME1 and PME48) have shown the importance of their functions in regulating the level of HG methylesterification during pollen germination and pollen tube growth (Jiang et al., 2005; Tian et al., 2006; Leroux et al., 2015). Mutation in one of the three genes induced severe retardation of pollen germination, pollen tubes with larger diameters that lead to high levels of burst tubes in the sub-apical dome. *VGD1* and *PME48* are the two most expressed genes in pollen (Leroux et al., 2015). In the phylogenetic tree generated by McCarthy et al. (2014), it appears that *VGD1* and *PME48* are not closely related to *PpPME*s. Moreover, silencing of a putative PME, the *Brassica campestris* Male Fertility 23a (BcMF23a), resulted in part in an impaired pollen tube growth *in vivo* (Yue et al., 2018). Recently, *ZmGa1P* (a pollen expressed PME) with another pollen PME (*Zm*PME10-1) localized at the tube tip was involved in preventing incompatibility response in maize (Zhang et al., 2018), revealing that PMEs can be implicated in different biological processes such as cell wall remodeling, incompatibility, response to biotic and abiotic stresses… (Le Gall et al., 2015; Fan et al., 2017; Wu et al., 2018) .

Moreover, moderate addition of exogenous commercial PME or pectinase has an effect on pollen tube (Parre and Geitmann, 2005b). Moderate concentration of pectinase was able to stimulate pollen tube growth whereas high concentrations promoted tip-swelling and bursting. This treatment was correlated with a reduced stiffness and increased visco-elasticity of the cell wall (Parre and Geitmann, 2005b). Furthermore, addition of kiwi PMEI in the culture medium induced significant pollen tube burst in the subapical region of the tip (Paynel et al., 2014). Interestingly, addition of recombinant *At*PMEI4 or *At*PMEI9 (which have different abilities to inhibit PME activity depending on the pH) to the culture medium had different effects on *A. thaliana* pollen tube growth. *At*PMEI4, like kiwi PMEI, induced a higher level of burst tubes in the sub-apical dome whereas *At*PMEI9 was promoting pollen tube growth (Hocq et al., 2017). All these data revealed that a strict balance between methylesterification/de-methylesterification of HGs is needed to maintain the mechanics of the cell wall and that local pH changes in the cell wall can influence this balance.

Wallace and Williams (2017) have identified 16 *AtPME1*-like homologs in *Nymphea odorata*. In addition, *VGD1*-like homologs were also more abundantly expressed in pollen grains and pollen tubes than in vegetative tissues of *N. odorata* (four *VGD1*-like homologs), and in *Amborella trichopoda* (one *VGD1*-like homolog) (Figure 1). The authors concluded that the finding of homologs of *VGD1* (PME with PMEI domain) which control the de-esterification at the tip in early-divergent angiosperms like *N. odorata* and *A. trichopoda*, is conserved among angiosperms. This event might be of importance in the development of fast-growing pollen tubes where refined control of PME activity near the tube tip is necessary. In contrast, none of the 10 *Pinus taeda* putative pollen/pollen tube-expressed PMEs suspected to be from group 2 PMEs were in the same clade than *AtVGD1* and 8 other *At*PMEs from group 2, suggesting that PMEs expressed in gymnosperm pollen tubes belong to the group 1 (i.e., without PMEI domain) (Wallace and Williams, 2017). It may explain a less clear de-esterification process of HGs in the slow-growing gymnosperm pollen tubes (Wallace and Williams, 2017). As mentioned above, it may not be the same in *P. meyeri* as pollen tubes grow 22-fold faster than *P. taeda* and display a clear zonation between highly (at the tip) and weakly (in the shank) methylesterified HGs (Chen et al., 2007).

Very little attention has been paid on pectin acetylesterases (PAEs) in tip-growing cells. The study on *PtPAE1* from *P. trichocarpa* over-expressed in *N. tabacum* induced a reduction of acetylation in the cell wall of pollen grains and a strong male sterility (Gou et al., 2012). Exogenous treatment with the recombinant *Pt*PAE1 on wild type pollen tubes displayed shorter and larger tubes (Gou et al., 2012) revealing that the control of de-esterification (methyl or acetyl) is impacting the mechanical properties of the cell wall.

PGs can hydrolyze weakly methylesterified HGs. This is of main importance especially in tip-growing cells that require a high remodeling capability, to allow weakening or strengthening of the cell wall. The *P. patens* genome contains 10 PG genes (McCarthy et al., 2014). The gene duplication during evolution is reflected by the increasing number of predicted genes: 16 in the lycophyte *S. moellendorffii*, 44 in the monocot *O. sativa*, 75 in the eudicot *P. trichocarpa* (Yang et al., 2013), and 67 in *A. thaliana* (McCarthy et al., 2014). Among them, 6 are predicted to be expressed in *A. thaliana* pollen (Mollet et al., 2013). The study of *Brassica campestris Male Fertility 26a (BcMF26a)* and *BcMF26b*, revealed in the double mutant that pollen tubes could not expand normally with occasional bursting in the sub-apical dome. They displayed larger diameters compared to the wild-type (Lyu et al., 2015), revealing that PGs are also important for normal tip-growth. Again, very little information is available on pectate lyases-like (PLLs) in tip-growing cells. The *P. patens* genome is predicted to contain 7 PLL genes, while 3 PLLs are expected in *S. moellendorffii* and very low numbers are predicted in monocots as well: 6 in *O. sativa* and 6 in *Sorghum bicolor* compared to eudicot plants (e.g., 26 in *A. thaliana*) (McCarthy et al., 2014).

A recent short communication has highlighted the possible role of PLLs in promoting pollen germination (Chebli and Geitmann, 2018). Interestingly, a previous report has shown that a pollen-specific calmodulin-binding protein, *No Pollen Germination 1* (*NPG1*), was required for pollen germination (Golovkin and Reddy, 2003). The same group reported that the N-terminal domain of this calmodulin-binding protein was able to interact with PLLs in the cell wall of pollen grains and growing pollen tubes suggesting that NPG1, may modulate PLL activity *via* calcium-calmodulin signaling, and control the HG modification in the expanding pollen tubes (Shin et al., 2014). These findings are particularly relevant because they outline a possible mode of molecular communication between the pollen tube and the female counterpart *via* calcium flux and calmodulin sensor which could regulate the fine tuning of pectin remodeling at the tip of the pollen tube (Steinhorst and Kudla, 2013).

### 4.2. Xylogalacturonan

XylGalA has a galacturonan backbone with substituting Xyl residues (Figure 1). XylGalA probed with LM8 (Supplemental Table 1) has been detected in the entire *A. thaliana* pollen tube (Dardelle et al., 2010). It seems that XylGalA is not present in *P. patens* protonemal cells, as no signal was observed with the same mAb (Moller et al., 2007). Moreover, out of 16 gene families involved in pectin synthesis and remodeling analyzed by McCarthy et al. (2014), orthologs of *A. thaliana* XylGalA xylosyltransferase genes were not found in the genome of *P. patens*, suggesting that XylGalA appeared later. Unfortunately, no information is currently available on XylGalA of *C. richardii* or gymnosperm pollen tubes. In *A. thaliana*, the study of *xylogalacturonan deficient 1* (*xgd1*) which gene codes a XYLOSYLTRANSFERASE involved in XylGalA biosynthesis did not display peculiar phenotype on pollen grains or pollen tubes (Jensen et al., 2008), suggesting that this gene is probably not expressed in pollen.

### 4.3. Rhamnogalacturonan-I

#### 4.3.1. Distribution and Composition

One of the widely common features present in RG-I (Figure 4) are galactan, arabinogalactan and arabinan side chains in vascular plants. Despite the fact that McCarthy et al. (2014) did not find any RG-I arabinosyl transferase in *P. patens*, the typical linkages found in RG-I (2,4-Rha, 5-Ara, 4-Gal, 4,6-Gal) were found in protonemal cells but at very low levels (Moller et al., 2007), suggesting that RG-I is not a major motif. Similarly, CoMPP has revealed very weak labeling with LM5 (Supplemental Table 1), suggesting that RG-I has low levels of galactan branching side chains. This was also observed in rhizoids of *P. patens* (Berry et al., 2016) and *C. richardii* (Eeckhout et al., 2014). The labeling of galactan was also very weak in *P. wilsonii*, mostly localized in the distal end of the tube (Chen et al., 2008). *A. thaliana* pollen tubes contain also low levels of 4-Gal and 4,6-Gal (Dardelle et al., 2010) and in *N. alata* pollen tubes, none of these linkages were found (Lampugnani et al., 2013). All these data suggest a common feature, that galactan side chains may not be an important structural component in those tip-growing cells. In contrast, a strong labeling was detected in *P. patens* protonemata with LM6 (Berry et al., 2016) and CoMPP (Moller et al., 2007). However, linkage analysis revealed very low level of 5-linked Ara*f* suggesting that arabinan side chains of RG-I were also in low amount and that LM6 may have bind to other polymers, such as AGPs (Moller et al., 2007), which are abundant in the cell wall of *P. patens* protonemata (Lee et al., 2005). Again, a strong labeling was also observed in the whole rhizoids of *P. patens* (Berry et al., 2016) and *C. richardii* (Eeckhout et al., 2014). Similar labeling pattern was observed in *A. thaliana* pollen tubes probed with LM6 and LM13 (Dardelle et al., 2010). A more diffuse labeling was detected in the entire pollen tube of *P. wilsonii* (Chen et al., 2008).

In contrast with *P. patens*, the level of 5-Ara*f* is important in pollen tube cell walls (Dardelle et al., 2010; Lampugnani et al., 2013) revealing another important difference between these tip-growing cells. However, it has been suggested in *N. alata* pollen tubes, that (1,5)-α-arabinans were not part of the side chains of RG-I but were considered free linear α-(1-5)-arabinan (Lampugnani et al., 2016). It may also be the case in *A. thaliana* pollen tubes, as the level of Rha is very low like in *N. alata*: 5 and 1%, respectively (Dardelle et al., 2010; Lampugnani et al., 2013) . The function of this free arabinan in *N. alata* pollen tubes is unknown but it may have an important role during pollen tube growth within the female tissues. Unfortunately, no immunolocalization of arabinans or linkage analysis has been performed on gymnosperm and commelinid pollen tubes. Thus, we cannot conclude that this high level of arabinosylation is a general feature of pollen tubes or only appeared in fast-growing pollen tubes.

#### 4.3.2. Biosynthesis

Orthologs of *A. thaliana* RG-I Ara transferase genes were not found in the genome of *P. patens*, whereas one ortholog was identified in the genome of *S. moellendorffii* (McCarthy et al., 2014). This lack of biosynthesis enzymes explains in part the very low level of arabinans in protonemal cells of *P. patens*. A study has highlighted the function of *N. alata* ARABINAN DEFICIENT-LIKE1 (*Na*ARADL1) related to ARABINAN DEFICIENT1 (*At*ARAD1) in pollen tubes (Lampugnani et al., 2016). This study revealed that the protein is resident in the Golgi apparatus. When the gene is introduced in *A. thaliana*, the cell wall exhibits more arabinans and the plant synthesizes a guttation fluid composed of soluble linear (1,5)-α-arabinans suggesting that arabinans are not side chains of RG-I (Lampugnani et al., 2016). Finally, REVERSIBLY GLYCOSYLATED PEPTIDEs (RGPs) involved in the interconversion between UDP-Ara*p* and UDP-Ara*f* was shown to be involved in pollen formation through the study of a *rgp1/2* double mutant (Drakakaki et al., 2006).

### 4.4. Rhamnogalacturonan-II

#### 4.4.1. Distribution and Composition

RG-II (Figure 4) contains unusual sugars like 2-keto-3deoxy-D-lyxo-heptulosaric acid (Dha) or 2-keto-3-deoxy-D-manno-octulosonic acid (Kdo) the latter is also found in bacteria (Smyth and Marchant, 2013). In plants, these sugars are only found in RG-II (Dumont et al., 2016). RG-II with the typical side chains has not been described in *P. patens* despite the fact that mosses possess putative homologs of CMP-Kdo-synthase genes. They are able to synthesize UDP-apiose (Smith et al., 2016) and sugars found in RG-II such as methylated Xyl and Fuc (Matsunaga et al., 2004; Roberts et al., 2012). However, RG-II-like structures have been described in the gametophyte of several bryophytes, but in quite low quantities, representing only 1% of what is found in angiosperm cell walls. Thus, the abundance of RG-II in the cell wall seems to have increased with increasing complexity of plants during evolution. Thus, it may suggest that RG-II is not as important in mosses compared to vascular plants (ferns, gymnosperms and angiosperms) (Figure 1) (Matsunaga et al., 2004). The study on the composition and distribution of RG-II has been a challenge in tip-growing cells due to the lack of mAb and the difficulty to collect sufficient cell wall material. However, Dumont et al. (2014) were able to biochemically detect Kdo in *A. thaliana* pollen tubes and using a polyclonal antibody. RG-II was weakly detected in the pollen tube cell wall of *L. longiflorum* (Matoh et al., 1998), *A. thaliana, Nicotiana benthamiana* and *Solanum lycopersicum* (Dumont et al., 2014). The question that remains to answer is: does RG-II exist also as boron-mediated dimers in the cell wall of tip-growing cells?

#### 4.4.2. Biosynthesis

In *A. thaliana* pollen tubes, six genes involved in RG-II synthesis have been described so far, and were shown to be important in the tip-growth. KDO-8-P SYNTHASEs (AtKDSA1 and AtKDSA2) are involved in the synthesis of Kdo. *Atkdsa1/Atkdsa2* double mutant was unable to form an elongated pollen tube and to perform fertilization (Delmas et al., 2008). CTP:Kdo cytidylyltransferase (CMP:Kdo Synthetase, CKS) activates Kdo as a nucleotide sugar. Mutation in *CKS* was investigated by Kobayashi et al. (2011) and induces the inhibition of pollen tube elongation. Two *male gametophyte defective* (*mgp*) mutants were isolated: *mgp4*, KO for a RG-II XYLOSYLTRANSFERASE (Liu et al., 2011) and *mgp2*, KO for a CMP-SIALYLTRANSFERASE-LIKE (Deng et al., 2010). The encoded protein for the last gene was suspected to transfer Dha or Kdo, as sialic acid has not been described in plants so far. The two resulting mutants display a strong delay of pollen tube growth. The other SIA2 (SIALYLTRANSFERASE-LIKE2) was also shown to be involved in the pollen tube growth as mutation in *SIA2* displayed a decrease in pollen germination and cell wall abnormalities (dichotomous branching tips or swollen tubes) (Dumont et al., 2014). Finally, in tomato, silencing of two GDP-D-Man epimerases which convert GDP-D-Man to GDP-L-Gal, key enzymes in ascorbate and cell wall biosynthesis resulted in the RNAi-SlGME1 line in pollen development defect and fruit size reduction and in the RNAi-SlGME2 line an alteration of RG-II dimerization, suggesting a sub-functionalization depending on the tissue (Mounet-Gilbert et al., 2016). All the data suggest an important role of RG-II in the tip-growth of pollen tubes. However, further investigations are necessary on other biological models.

## 5. Hydroxyproline-Rich GlycoProteins (HRGPs)

HRGPs are divided in two families: highly *O*-glycosylated arabinogalactan proteins (AGPs) and moderately *O*-glycosylated extensins (Showalter and Basu, 2016; Johnson et al., 2017a).

### 5.1. Arabinogalactan-Proteins

**Figure 5:**
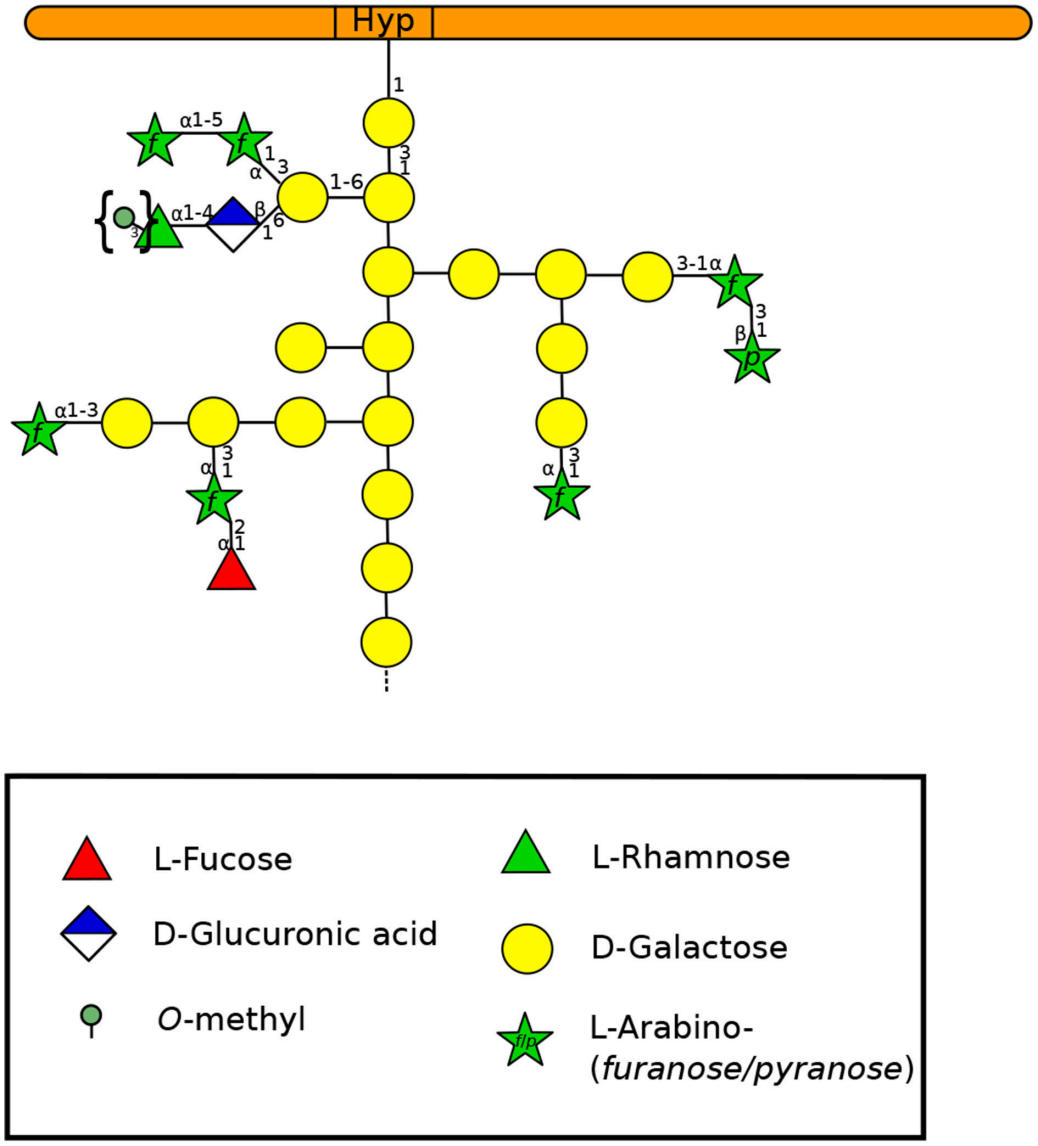
Type-II arabinogalactan structure of a classical AGP according to Nguema-Ona et al. (2012) and Showalter and Basu (2016). Methylesters in braces have been described in mosses and ferns (Fu et al., 2007; Moller et al., 2007; Bartels and Classen, 2017). A GlyceroPhosphatidyl Inositol (GPI) anchor can be found in the N-terminal region of the protein but is not represented here. Monosaccharides are represented according to the Symbol Nomenclature for Glycans (SNFG) (Varki et al., 2015).

AGPs are found throughout the entire plant kingdom from bryophytes (Lee et al., 2005) to angiosperms (Seifert and Roberts, 2007; Ellis et al., 2010; Ma et al., 2017). They have been involved in a large number of biological functions including tip-growth (Nguema-Ona et al., 2012). The protein backbone of AGPs is extensively *O*-glycosylated accounting for more than 90% (w/w) of the mass of the molecule (Figure 5). AGPs consist of a type-II arabinogalactan with a β-(1,3)-galactan backbone and both β-(1,6)-galactosyl and α-(1-5)-arabinosyl residues (Figure 5). They also possess some less abundant sugars including Xyl, Fuc, Rha, and GlcA (Nothnagel, 1997; Showalter, 2001; Tan et al., 2010; Nguema-Ona et al., 2014). Despite its low level, GlcA was shown to bind calcium and thus, AGPs could be an external source of Ca^2+^ necessary for the intracellular machinery of pollen tube growth including Golgi vesicle exocytosis (Lamport et al., 2014). AGPs may also play a role in plasticizing the cell wall (Lamport et al., 2018). In bryophytes such as *Sphagnum sp., Polytrichastrum formosum* and *P. patens*, and the monilophytes like *Equisetum arvense, Dryopteris filix-mas* and *Pteridium aquilinum*, the typical type-II arabinogalactan linkages found in AGPs were detected (t-Ara*f* , 4-GlcA*p*, 3-Gal*p*, 3,6-Gal*p*). However, unusual sugars were also analyzed such as a terminal 3-*O*-methyl-L-Rha in ferns and mosses which has not been found in angiosperms so far (Fu et al., 2007; Moller et al., 2007; Bartels and Classen, 2017), or even 1,2,3-linked Gal residues never described before in AGPs (Bartels and Classen, 2017). Interestingly, in the lycophytes like *Lycopodium annotinum*, the terminal 3-*O*-methyl-L-Rha was not detected. Instead high levels of t-Ara*p* were found (Bartels and Classen, 2017). In *A. thaliana* pollen tubes, one study has highlighted the presence of linkages that can be found in AGPs such as t-Ara, 3-Gal and 3,6-Gal (Dardelle et al., 2010). Similar results were obtained in *N. alata* pollen tube cell walls with the detection of t-Ara*f* , t-Ara*p*, 3-Gal*p*, and 3,6-Gal*p* (Lampugnani et al., 2013).

AGPs are classified into 9 families based on the protein backbone structure: classical AGPs, AG peptides, fasciclin-like AGPs (FLA), Lys-rich AGPs, early nodulin- (ENOD) like AGPs, non-specific lipid transfer protein-like AGPs (nsLTP-like AGPs), phytocyanin-like AGPs, xylogen-like AGPs and chimeric AGP-extensins (Ma and Zhao, 2010; Showalter et al., 2010; Tan et al., 2012; Johnson et al., 2017a; Ma et al., 2017). Among them, some can be anchored to the plasma membrane via GlyceroPhosphatidyl insositol (GPI) (Youl et al., 1998; Svetek et al., 1999). Classical AGPs, AG-peptides, FLAs, phytocyanins, and lipid transfer-like proteins are among the GPI-anchored proteins identified in *A. thaliana* pollen tubes by transcriptomic and proteomic analyses (Lalanne et al., 2004). The importance of GPI-anchored proteins was highlighted by the disruption of *SETH1* and *SETH2* genes that encode subunit homologs of a GPI-N-acetylglucosaminyltransferase (GPI-GnT) complex involved in GPI anchor synthesis (Lalanne et al., 2004). The mutant plants display a strong reduction of pollen germination and pollen tube growth, as well as abnormal callose deposition possibly by affecting simultaneously a large number of GPI-anchored proteins. Transcriptome data analyses suggested that GPI anchored AGPs were already quite abundant in plant lineages such as mosses and liverworts (Johnson et al., 2017b). The question remains however to what extent the GPI-anchor of a protein affects its function (Nguema-Ona et al., 2012). Using a bioinformatics approach, Ma et al. (2017) have predicted that classical AGPs, AG-peptides, and Lys-rich AGPs first emerged in *P. patens, S. moellendorffii* and the gymnosperm *P. abies*, respectively. They also revealed that the number of predicted genes varied greatly across plant lineages: 104 in the bryophyte *P. patens*, 49 in the pteridophyte *S. moellendorffii*, 129 in the gymnosperm *P. abies*, 48 in the basal angiosperm *A. trichopoda* (Figure 1) (Amborella Genome Project, 2013), in monocots between 82 in the orchid *Phalaenopsis equestris* and 305 in *Zea mays* and in eudicot plants between 77 in *Carica papaya* and 313 in *Glycine max* (Ma et al., 2017).

Several mAbs and reagents have been used to localize AGPs (Table 2; Supplemental Table 1). Among them, the β-D-Glucosyl Yariv (βGlcY), able to bind and precipitate AGPs (Yariv et al., 1967), was used to study protonemal expansion of *M. polymorpha* (Shibaya et al., 2005), *P. patens* (Lee et al., 2005) and pollen tube growth in several species including the angiosperm Magnoliales: *Asimina triloba*, the monocot *L. longiflorum* or the eudicots *N. tabacum, Malus domestica* and others (Figure 1) (Mollet et al., 2002; Nguema-Ona et al., 2012; Fang et al., 2016b; Losada et al., 2017). *N. tabacum* pollen tube growth was not affected by a treatment with βGlcY and AGPs were not detected at the tip using several mAbs such as MAC207 and JIM8 (Supplemental Table 1). However, a ring-like pattern was observed in the shank of the tube after cellulase or pectinase treatments (Li et al., 1992).

**Table 2:** AGP localization in expending tip-growing gametophytes (rhizoid, protonema and pollen tube) grown *in vitro* across several plant lineages.

**Species**	**Probe**	**Cell type**	**Specimen treatment**	**Labeling pattern/effect**	**References**
**Bryophytes**					
*Marchantia polymorpha*	βGlcY	Protonema	Living protonemata	- Block protonemal initiation	Shibaya et al., 2005
*Physcomitrella patens*	LM6	Protonema	Cell surface	- Strong at the type	Lee et al., 2005
			Sections	- Strong at the tip and the whole cells	
	βGlcY	Protonema	Living protonemata	- At the tip with tip growth arrest	
	LM2, JIM13	Rhizoid	Cell surface	- LM2 : weak on the whole rhizoid	Berry et al., 2016
				- JIM13 : none	
**Monilophytes**					
*Ceratopteris richardii*	βGlcY	Rhizoid	Living rhizoids	- Whole rhizoid	Lopez and Renzaglia, 2014
	LM2, LM6	Rhizoid	Cell surface	- Strong on the whole rhizoid	Eeckhout et al., 2014
Angiosperms^*^					
**Magnoliales**					
*Asimina triloba*	βGlcY	Pollen tube	Living pollen tubes	- Tip and spots in the shank, with growth reduction	Losada et al., 2017
**Eudicots**					
**Ranunculales**					
*Aquilegia eximia*	βGlcY	Pollen tube	Living pollen tubes	- No labeling and no growth arrest	Mollet et al., 2002
**Rosales**					
*Malus domestica*	LM2	Pollen tube	Cell surface	- Decreasing intensity from the pollen grain to the tip	Fang et al., 2016a
**Solanales**					
*Lycopersicum pimpinellifolium*	βGlcY	Pollen tube	Living pollen tubes	- No labeling and no growth arrest	Mollet et al., 2002

* *For additional informations on gymnosperm and other angiosperm pollen tube species not listed here, see Nguema-Ona et al. (2012)*.

On the other hand, a treatment with βGlcY strongly reduced or arrested the tip expansion of *P. patens* protonemata (both caulonemal and chloronemal cells) (Lee et al., 2005), the growth of *M. polymorpha* protonemata (Shibaya et al., 2005) or pollen tubes that displayed AGPs at their tips (Mollet et al., 2002; Leszczuk et al., 2019) and in the shank as a periodic ring-like deposition after a pectinase treatment (Jauh and Lord, 1996) (Table 2). In lily and strawberry (*Fragaria* x *ananassa*) pollen tubes and in *P. patens* protonemata, the growth arrest was fast but reversible after removal of the reagent. In the case of lily pollen tubes, despite the growth arrest, vesicular secretion at the tube tip was persisting. Disorganized cell wall architecture and composition were observed due to an abnormal callose deposition (Roy et al., 1998; Leszczuk et al., 2019). This change of cell wall composition and organization in the tip induced a change in the mechanical properties of the cell wall, being stiffer in the βGlcY treated pollen tube tip and even more in the new emerged pollen tube compared to the untreated control (Leszczuk et al., 2019). The authors suggested that this hardening may be related to the appearance of weakly methylesterified HG in the tip of the newly-grown pollen tube. Similarly, an abnormal cell wall deposition was also observed at the tube tip of *M. polymorpha* protonemata treated with βGlcY (Shibaya et al., 2005). The main difference between both tip-growing cells is that the protonema arrested-tip slowly resumed growth 3h after the removal of βGlcY (Lee et al., 2005) whereas in lily and strawberry pollen tubes, the arrested-tips did not resume growth but a new tip emerged back from it (Mollet et al., 2002,Leszczuk et al., 2019).

Inactivation of *P. patens* AGP1 encoding a classical AGP resulted in reduced cell expansion suggesting its role in tip-growth (Lee et al., 2005). In *A. thaliana*, four genes: two classical GPI anchored AGPs (AtAGP6 and AtAGP11) and two AG-peptides (AtAGP23 and AtAGP40) are expressed only in pollen grains and pollen tubes. The double mutant *agp6/11* has reduced pollen tube growth and seed set (Levitin et al., 2008; Coimbra et al., 2010) indicating that AGPs are important during pollen tube growth. Using yeast two-hybrids, interactors of AGP6 and AGP11 were mostly involved in recycling of cell membrane components *via* endocytosis (Costa et al., 2013). It revealed that endosomal trafficking pathways are fundamental regulators of tip-growing cells. In a search of pollen specific *AtAGP6/11* orthologs from 1,000 plant transcriptomes, Johnson et al. (2017b) revealed that they could be confidently detected in the basal angiosperms (*A. trichopoda*), basal eudicots (Ranunculales), Rosids (Brassicales, Fabales, Myrtales), Asterids (Apiales, Solanales, Asterales, Ericales), the non-commelinid (Liliales, Arecales, and Asparagales) and commelinid (Poales including rice) monocot lineages, suggesting the existence of an ancestral gene (Figure 1). In rhizoids of *P. patens*, labeling with LM2 and JIM13 was either weak on the entire rhizoid or absent (Figure 2) (Berry et al., 2016). In contrast, the labeling of AGPs (with LM2 and LM6) (Supplemental Table 1) was very strong on the whole rhizoid of the monilophyte *C. richardii* (Table 2) (Eeckhout et al., 2014), suggesting either a different carbohydrate composition of AGPs and/or a different accessibility of the mAb to the epitopes thus supporting a different cell wall organization. Cell-surface immuno-labeling or βGlcY coloration revealed a strong labeling at the tip in the protonemata of *M. polymorpha* and *P. patens* and pollen tubes from lily (Mollet et al., 2002), *Asinima* (Losada et al., 2017) and *A. thaliana* (Dardelle et al., 2010). In gymnosperms like *P. wilsonii* or *P. meyeri*, labeling with LM2 was located in the entire pollen tube wall or as a periodic ring-like pattern (Chen et al., 2007, 2008). With the same mAb, a ring-like pattern was also observed in the cell wall of apple pollen tubes (Table 2) (Fang et al., 2016a).

### 5.2. Extensins

**Figure 6:**
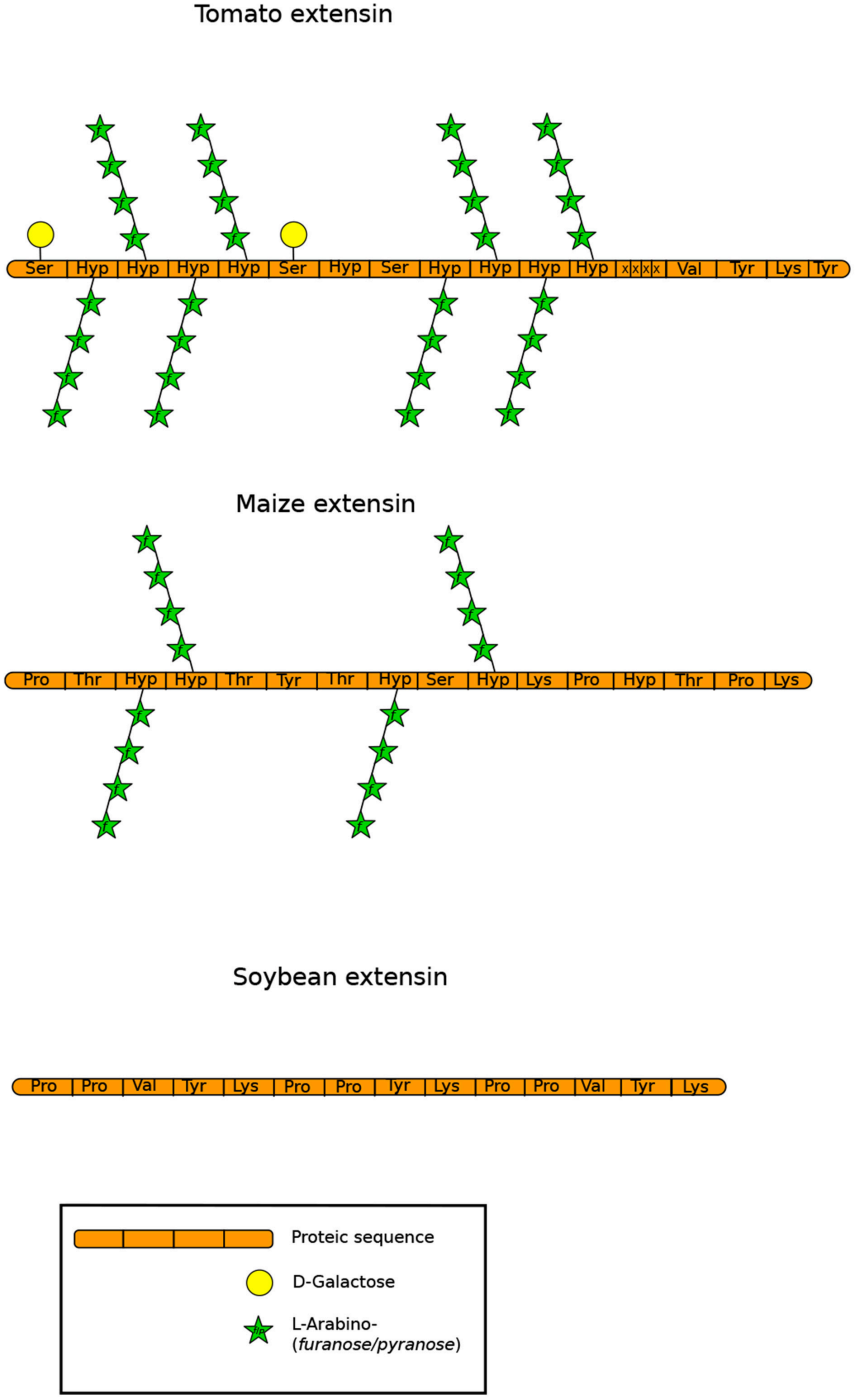
Structures of extensins described by Carpita et al. (2015) and Showalter and Basu (2016) showing different levels of *O*-glycosylation. Monosaccharides are represented according to the SNFG (Varki et al., 2015).

Extensins (EXTs) are repeated pentapeptide Ser(Hyp)_4_ that are moderately *O*-glycosylated with short Ara and Gal oligosaccharides on Hyp and Ser residues respectively (Figure 6) (Kieliszewski and Lamport, 1994; Velasquez et al., 2015). In the cell wall, EXTs play an important role in development, and cross linking of EXTs is generally associated with the arrest of cell expansion (Johnson et al., 2017b). The cross-linked EXTs were predicted to occur in most land plants except in commelinid monocot grasses and arose in non-vascular plants, particularly in hornworts (Dendrocerotales, Notothyladales…). They were less represented in liverworts (Marchantiales…) and mosses (Funariales including *P. patens*, Polytrichales, Sphagnales…). Indeed, classical EXTs are predicted to be absent from *P. patens* as well as from the gymnosperm *Pinus taeda* (Liu et al., 2016; Johnson et al., 2017b). Accordingly, using microarrays, almost no detectable signal was observed with LM1, JIM19 and JIM20 (Supplemental Table 1) in fractionated cell wall extracts from *P. patens* protonemata (Moller et al., 2007). Similarly, no detectable signal was observed with LM1 in *A. thaliana* pollen tubes (Dardelle et al., 2010) suggesting either a very low abundance, epitope masking or different carbohydrate epitopes. In the spikemoss, *S. moellendorffii*, very weak signal was also observed with LM1 but clear signal was detected with JIM20 on an alkali-extracted cell wall extract (Harholt et al., 2012).

Non-classical EXTs include short EXTs and chimeric proteins such as LRX (Leucine-Rich repeat EXT), proline-rich EXT-like receptor kinases (PERKs), formin-homolog EXTs (FH EXTs) (Liu et al., 2016), AGP-EXTs, which are highly represented in gymnosperms (conifers, gnetophytes, *Ginkgo* and Cycadales) (Johnson et al., 2017b) and others. LRXs, PERKs and FH EXTs derived earlier than classical EXTs and were predicted to be present also in *P. patens* and *S. moellendorffii* (Liu et al., 2016).

Genes involved in EXT glycosylation such as β-arabinosyltransferases of the GT77 family were found in *S. moellendorffii* and *P. patens* (Harholt et al., 2012). The importance of the *O*-glycosylation status of EXT on the conformation of the glycoprotein has been described (Stafstrom and Staehelin, 1986) and was further investigated by functional genomics on the tip-growing protonemata of *P. patens* and *A. thaliana* pollen tubes (MacAlister et al., 2016). Mutations on genes coding the enzymes responsible for initiating oligoarabinose chains on the protein backbone, the hydroxyproline *O*-arabinosyltransferases (HPATs) (Ogawa-Ohnishi et al., 2013) revealed opposite effects on those two models (MacAlister et al., 2016). *Athpat1/3* double mutant pollen tubes showed both *in vivo* and *in vitro* a strong reduction of pollen tube expansion. In contrast, among the two genes in *P. patens* (*HPATa* and *HPATb*), mutation in *HPATa* displayed the most striking phenotype by inducing an increase in the number and length of filaments which was correlated with a faster growth of the protonema in the *hpata/b* double mutant than in the wild type. Interestingly, the double mutant did not display any defects on the length of the other tip-polarized cells: the rhizoids. This suggests that other *HPAT* genes are expressed in this structure. The authors suggested that the different responses between the two models may be related to the growth rates (which are faster in pollen tubes), the cell wall compositions (which are quite different), or the different target proteins of these enzymes (MacAlister et al., 2016).

Another study showing the important function of EXTs in pollen tube growth was conducted on the classical EXT18 (Choudhary et al., 2015). Despite pleiotropic phenotypes, mutation in *EXT18* reduced significantly the pollen viability, pollen tube growth and increased the level of burst tubes leading to a strong reduction of seed set (Choudhary et al., 2015). Moreover, the level of expression of 12 other classical EXTs was modified in the mutant. Two were up-regulated, including *EXT19* (closely related to *EXT18*), and ten were down-regulated, suggesting a coordinated network of expression between the *EXT* genes (Choudhary et al., 2015).

Evidence of the presence of LRX in pollen tubes was highlighted in maize more than 20 years ago by immunolocalization of PEX1 (Pollen EXT1) in the inner callosic cell wall layer (Rubinstein et al., 1995). *PEX*s were then also found in other monocots (Sorghum, rice, Brachypodium…) and eudicots (tomato, *Arabidopsis, Brassica rapa*, potato…) (Stratford et al., 2001; Liu et al., 2016). More recently, three studies have shown the important role of *LRX* in pollen tube growth and cell wall mechanics (Fabrice et al., 2018; Sede et al., 2018; Wang et al., 2018). In *A thaliana*, among the 11 LRX genes, four of them (*LRX8-11*, previously described as *PEX1-4*) are devoted to the reproductive development (Baumberger et al., 2003). The LRR domain is thought to bind an interaction partners which have been recently characterized as the pollen secreted signaling peptides RALF4 and RALF19 (RAPID ALKALINIZATION FACTOR). The two peptides maintain the pollen tube cell wall integrity and are translocated into the interior of the pollen tube via the CrRLK1L (*Catharanthus roseus* Receptor-Like Kinases1-Like subfamily (Mecchia et al., 2017) while the EXT domain anchors the protein in the cell wall (Ringli, 2010), reviewed by Marzol et al. (2018). Interestingly, abnormally short and burst rhizoid structures were also observed in *M. polymorpha* mutants defective in *MpTHESEUS* that belongs to the *CrRLK1L* family (Honkanen et al., 2016). These data reveal that cell wall integrity sensing is conserved in tip-growing of modern seedless land plants (Honkanen et al., 2016).

The studies from single, to quadruple mutants in *LRX8-11* revealed a weak phenotype for the single mutants but severe in the double *lrx8/9*, and the triple *lrx8/9/10, lrx8/9/11*, or *lrx9/10/11* mutants. It was observed a strong decrease of pollen germination, an increased level of bursting and abnormal tubes. Such mutants show wavy growth, widened tips, leakage in the sub-apical region of cytoplasmic contents enriched in cell wall polymers. It was observed the emergence of a bulge at the shank or at the pollen tube tip which causes tip bursting leading *in vivo* to a strong reduction of seed set (Fabrice et al., 2018; Sede et al., 2018; Wang et al., 2018). In those mutants, pollen tubes displayed abnormal cell walls with altered levels of pectins including HG and RG-I, fucosylated XyG and accumulation of callose (Fabrice et al., 2018; Sede et al., 2018; Wang et al., 2018). These defects in the cell wall composition and organization led to an increase of internal turgor pressure and cell wall stiffness of the pollen tube (Fabrice et al., 2018). Interestingly, the pollen tube growth defects observed in *lrx8/9/11* triple mutant, were partially supressed by decreasing the calcium concentration, or adding LaCl_3_, a calcium channel inhibitor. It suggests that LRX proteins influence Ca^2+^-related processes (Fabrice et al., 2018). Indeed, monitoring calcium level in the triple mutant revealed a very strong spike of calcium just before bursting (Fabrice et al., 2018).

All the data accumulated suggest that EXTs provide either structural support at the tip of the growing pollen tube and/or can serve as an intermediate in the communication between the cell wall and the cytoplasm (Bascom et al., 2018).

## 6. Callose

Callose is a linear plant polymer of β(1,3)-linked Glc with some β(1,6)-branches containing a small fraction (approximately 2%) of GlcA (Stone and Clarke, 1992). In flowering plants, callose is generally constitutively absent in the cell wall of somatic cells but appears during cytokinesis (Scherp et al., 2001), and in response to biotic and abiotic stresses. It can also be deposited in sieve plates and plamodesmata (Abel et al., 1989; Scherp et al., 2001; Tang, 2007; Chen and Kim, 2009). Wall permeability is thought to be largely controlled by callose which can be considered as a leak sealant (Parre and Geitmann, 2005a; Geitmann and Steer, 2006).

### 6.1. Callose in Protonemata and Rhizoids of Bryophytes and Pteridophytes

Little information is known about callose deposition in bryophytes and pteridophytes. However, cytochemical staining (Supplemental Table 1) have shown that callose was present in the aperture of developing *P. patens* spores and in protonemata of *P. patens* (Moller et al., 2007; Tang, 2007; Schuette et al., 2009; Berry et al., 2016) and *F. hygrometrica* (Bopp et al., 1991). In *P. patens*, the analysis of 3-Glc linkage confirmed the presence of callose (Moller et al., 2007). Callose deposition has also been observed to some extent in rhizoids of the liverwort *M. polymorpha* (Cao et al., 2014) and the C-fern *C. richardii* (Eeckhout et al., 2014). These different studies show that callose deposits constitute a permanent compound already present in spores and very probably associated to the development of both protonemata and rhizoids in bryophytes and pteridophytes.

### 6.2. Callose in Pollen Tube Cell Walls

Callose is found in the cell wall of pollen grains in gymnosperms such as *Ginkgo/cycads*, but not in their pollen tube cell walls (Yatomi et al., 2002; Abercrombie et al., 2011). Using aniline blue staining (Supplemental Table 1), callose was not detected in the pollen tube wall of several *Pinus* species but detected in *Podocarpus nagi* and *Chamaecyparis obtusa* pollen tube (Yatomi et al., 2002). In contrast, using a callose-specific mAb (Supplemental Table 1), the pollen tube cell walls of six tested *Pinus* species were labeled to various degrees: a slight labeling was observed in most of the examined conifers (Yatomi et al., 2002; Fernando et al., 2010), none in *Pinus taeda* (Abercrombie et al., 2011), but in the entire wall of two *Podocarpus* species, *Cryptomeria japonica* and *C. obtusa*. Callose is also uniformly distributed along the tube shank and a very faint fluorescence could be detected in the tip region of pollen tubes from *P. meyeri* (Chen et al., 2007). Similarly, callose was found transiently in the tip of *P. sylvestris* pollen tubes (Derksen et al., 1999). This tip-localized callose deposition is generally not observed in angiosperm pollen tubes grown *in vitro* in normal culture conditions (Mollet et al., 2013). The role of these transient callose deposits at the gymnosperm pollen tube tip is unknown. However, Pacini et al. (1999) suggested that callose observed at the pollen tip may not have a structural function but rather could serve as a reserve polysaccharide, although this hypothesis needs to be confirmed.

Interestingly, in the shank of gymnosperm pollen tubes studied so far, the cell wall is composed of one layer (Derksen et al., 1999; Yatomi et al., 2002; Fernando et al., 2010) whereas in angiosperms, two layers are present, an outer non-callosic cell wall layer and an inner callose-enriched layer (Meikle et al., 1991; Ferguson et al., 1998; Dardelle et al., 2010; Derksen et al., 2011; Chebli et al., 2012; Mollet et al., 2013). Consequently, in contrast with other cells callose is one of the main cell wall components of the pollen tube in angiosperms representing about 35% in 16-h old *A. thaliana* or *N. alata* pollen tubes, along with arabinans (Dardelle et al., 2010; Lampugnani et al., 2013) .

### 6.3. Callose Plugs in Pollen Tubes

An interesting difference between gymnosperms and angiosperms pollen tubes is a lack of callose plug formation in the gymnosperm studied, so far (Fernando et al., 2010; Abercrombie et al., 2011). In angiosperms, callose plugs are synthesized periodically to maintain the vegetative cell in the tip-region. These plugs are present in the early-divergent angiosperm *A. trichopoda* (Williams, 2009). Their functions is to seal off the pollen tube from the pollen grain and to separate the young from the older parts of the tube maintaining a manageable cytosolic volume. These callosic barriers reduce the risk of damage and allow the tube to grow longer distances (Taylor and Hepler, 1997). In the eudicot *Camellia japonica*, the average degree of polymerization (DP) of callose found in the plugs was at least 90 whereas in the inner pollen tube cell wall the DP was much lower (DP ≈ 21) suggesting a possible more cohesive structure of callose deposits in the plugs than in the cell wall (Nakamura et al., 1980, 1984). Within angiosperms, the morphology of callose plugs and the patterns of their depositions were previously shown to vary among species (Mogami et al., 2006; Qin et al., 2012). Variability in the plug position has also been observed in pollen tubes among ecotypes and even within an ecotype (Qin et al., 2012). In this study covering 14 *A. thaliana* ecotypes, it was demonstrated that the position of the first callose plug and subsequently the number of callose plugs within a pollen tube will have an impact on its length. Thus, pollen tubes with the first callose plug near the pollen grain were on average longer than those with the first callose plug farther from the grain. This pattern of callose plug deposition was heritable (Qin et al., 2012). This indicates that the periodic deposition of callose plugs can impact pollen tube length.

### 6.4. Callose Biosynthesis

Callose biosynthesis is catalyzed by a multi-subunit enzyme complex associated to the plasma membrane in numerous types of plant cells (Verma and Hong, 2001). The key components of this complex are grouped under the name of CALLOSE SYNTHASES (CalS) / GLUCAN SYNTHASE-LIKE (GSL) (Li et al., 2003; Farrokhi et al., 2006; Abercrombie et al., 2011), that encode GT48 β-(1-3)-glucan synthase enzymes (Tucker et al., 2018). The involvement of CalS in spore development is a plesimorphic feature of terrestrial plants. Callose is synthesized from UDP-Glc, that binds directly to the catalytic subunit of CalS/GSL (Drbkov and Honys, 2017). Twelve *CalS* have been identified in *A. thaliana* distributed in four clades (Richmond and Somerville, 2000; Verma and Hong, 2001). These first identified genes were crucial for further investigation of *CalS-like* orthologs in other land plant models. Among these 12 genes, *CalS5, CalS11*, and *CalS12* are devoted to pollen development (Chen and Kim, 2009). *CalS5* was suggested to be involved in the synthesis of the callose wall during pollen grain germination, pollen tube growth and callose plug formation (Nishikawa et al., 2005). Knockout mutations of *CalS5* did not perturb vegetative growth significantly but resulted in a male sterility (Dong et al., 2005). The mutant *cals5* displayed a complete loss of callose plugs in the growing pollen tubes (Dong et al., 2005). However, another study on a *cals5* mutant obtained by point mutations showed also defect in callose deposition but the pollen tube growth was normal (Nishikawa et al., 2005). The authors suggested that callose was important for pollen wall patterning but not for the tube growth (Nishikawa et al., 2005). This report sows doubts about the exact role of callose plugs in the pollen tube growth. It seems highly surprising, that mutant pollen tubes without callose plugs may have the same performance and grow similarly as wild-type pollen tubes *in vitro* and *in vivo*. This finding questions the exact role of callose in pollen tubes while this polymer appears to be an evident evolutionary hinge at the sight of the phylogenetic studies. Overexpression of *CalS5* under the *CaMV 35S* promoter in *A. thaliana* also causes abnormal callose deposition and resulted in the precocious pollen germination prior to anthesis, suggesting that the expression level and localization pattern of CalS5 are important for pollen development (Xie et al., 2010).

As in *A. thaliana*, the genome of *P. patens* contains also 12 putative *PpCalS* that cluster in three clades with *AtCalS* genes (Schuette et al., 2009). *PpCalS5*, being the ortholog of *AtCalS5*, was suspected to be involved in the spore development (Schuette et al., 2009). In 2011, a study on *CalS* expression patterns brought evidence that *AtCalS5* orthologs were expressed in mature pollen grains and pollen tubes of many different early-divergent angiosperms including the basal angiosperm *A. trichopoda*. In gymnosperms, *Ginkgo CalS5* and *Gnetum CalS5* were only expressed in pollen grains whereas *CalS5* orthologs were only expressed in fast-growing pollen tubes (i.e., angiosperms) suggesting that *CalS5* plays different but crucial roles during pollen formation and/or pollen tube growth (Abercrombie et al., 2011). Finally, an ortholog of *AtCalS5* was also found in *S. moellendorffii* while the gymnosperm *Pinus taeda* pollen expressed another ortholog PtCalS13 (Abercrombie et al., 2011).

*NaGsl1*, a *CalS* gene, is specifically and abundantly expressed in pollen grains of *N. alata* (Doblin et al., 2001). Levels of *NaGsl1* transcripts were high in mature pollen, suggesting that pollen grains are released with the appropriate transcripts for wall formation before germination. Transcript levels remained high during pollen tube growth (Doblin et al., 2001). Most *CalS* are expressed under the dependence of the bZIP transcriptor factor family as seen in *A. thaliana* (Drbkov and Honys, 2017). In tobacco pollen tubes, *NaCalS* was localized in the distal region and at the tip of the pollen tube and accumulated, driven by microtubules, at the plasma membrane where callose plugs are synthesized (Cai et al., 2011).

### 6.5. Callose Remodeling During Pollen Tube Growth

Genes involved in callose hydrolysis and recycling, putatively involved in the pollen tube wall remodeling, includes the β-(1,3)-glucan hydrolase genes (GH17 family). These genes are notably conserved across land plant species (Tucker et al., 2018). Exogenous application of lyticase, an enzyme able to degrade callose, on pollen tubes can increase the pollen tube diameter more drastically in the distal region. As a consequence, the stiffness decreased while viscoelasticity increased in the distal region of the tube (Parre and Geitmann, 2005a). In contrast, in the tip region where callose is not present, no such effect was observed (Parre and Geitmann, 2005a). In the angiosperm *L. longiflorum*, exo-glucanases were described to play an important role in the regulation of pollen tube elongation (Takeda et al., 2004).

### 6.6. Callose and Callose Plugs: Important Markers of the Evolutionary Success of Flowering Plants With Fast-Growing Pollen Tubes

Callose represents about 80% of the wall mass of the tube shank (Schlupmann et al., 1994) and the bulk of wall volume in most angiosperms (Williams et al., 2016). There is evidence that building amorphous callose-based wall found in angiosperms is faster and more energy efficient than elaborating a cellulose microfibril-based wall produced by gymnosperms (Kudlicka and Brown Jr, 1997; Williams, 2012). Early-divergent angiosperms like *A. trichopoda, Nuphar polysepala* or *Austrobaileya scandens* have pollen tubes with a secondary callose wall and produce callose plugs compared to gymnosperms. The apparition of these two features in early-divergent angiosperms combined with novel secretory carpel tissues (Williams, 2009) are probably the reason of the difference of growth rates. The fast rate of pollen tube growth is sustained in angiosperms by efficient exocytosis of new wall material at the tip or synthesis at the plasma membrane in a small region at, or just behind, the tube apex (Williams et al., 2016) and endocytosis of cell wall components are probably of importance. In the shank of the tube, the inner callose layer is essential for insuring an efficient polarized tip-growth and resistance to turgor pressure. All these studies have highlighted that short-lived and fast-growing pollen tubes associated with the apparition of an inner callose wall and callose plugs were the fundamental innovations that have trigger the ecological success of angiosperms.

## 7. Conclusions and Perspectives

During evolution, cell wall has to adapt to changing external factors in all tip-growing plant systems. These external signals originate from the atmosphere, the soil, water and biotic interactions for protonemata and rhizoids, or from female tissues for pollen grains and pollen tubes. However, little is known about the internal sensors perceiving these external cues.

**Figure 7:**
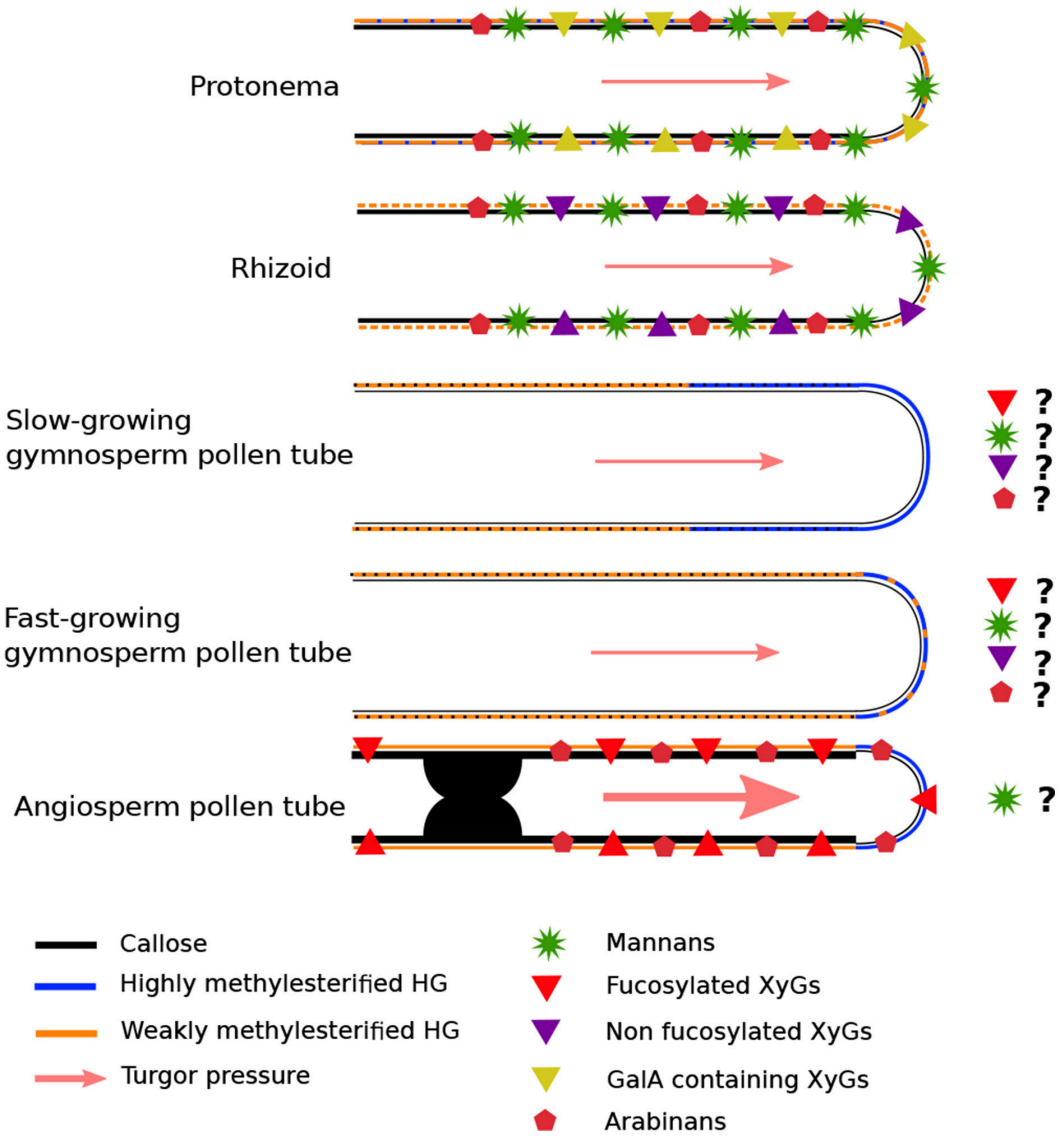
Model presenting the common and the different cell wall polymers found in gametophyte tip-growing cells. Protonemata and rhizoids illustrate the first slow tip-growing cells in *P. patens* with one cell wall layer composed of weakly methylesterified HGs, mannans, non-fucosylated and GalA containing XyGs and very low level of callose. Evolution has brought more specialized structure devoted to reproduction: pollen tubes. They display striking differences in growth speed: from slow-growing in some gymnosperms, moderate growth in other gymnosperms to fast-growing pollen tubes in angiosperms. In gymnosperms, only one cell wall layer and no callose have been observed. Due to the lack of information, a lot of question marks are shown. The cell wall contains callose, cellulose, and highly methylesterified HGs that is found in the tip and way behind it. In angiosperm monocot non-commelinid and eudicot pollen tubes, two cell wall layers are observed in the shank of the tube and a clear de-methylesterification of HGs is observed in the sub-apical dome. Callose plugs represented in black are regularly synthesized for maintaining the cell in the tip region of the pollen tube. The diameter differences are not in scale but depict variation between those structures. It was estimated based on published pictures. Cellulose is not represented.

Most of the polysaccharides found in the primary cell wall are present in gametophytic tip-growing cells throughout the land plant lineages. Cellulose, AGPs, XyGs and several pectic motifs including HGs and arabinans are detected in protonemata and pollen tubes (Figure 7). The very clear common point in all these gametophyte tip-growing cells is the abundance of arabinans, either being free in the cell wall, linked to RG-I or associated with AGPs. Arabinans were shown to be involved in cell wall flexibility (Jones et al., 2003; Moore et al., 2008; Verhertbruggen et al., 2013; Tenhaken, 2015) and many reports have shown that its modulation prevents water loss (Gribaa et al., 2013; Le Gall et al., 2015). However, differences are found. In rhizoids, the presence of HGs is unclear in the moss *P. patens* (Berry et al., 2016), but detected in *C. richardii* (Eeckhout et al., 2014). In contrast, mannans found in *P. patens* protonemata and rhizoids (Berry et al., 2016) (Figure 7), were not detected in rhizoids of the C-fern *C. richardii* and may have disappeared in the cell wall of eudicot pollen tubes. Today, mannans are mostly found in seeds and secondary cell walls of eudicot plants (Figure 7).

The co-existence of those two structures (cellulose and mannan) in *P. patens* may have help to sustain resistance to turgor pressure. Interestingly, in the fast-growing eudicot pollen tubes, mannans have not been found so far and cellulose became a minor component (in terms of quantity) compared to sporophyte cell walls.

During evolution, the first terrestrial plants possessed long-lived and multi-task tip-growing cells like protonemata and rhizoids that are involved in nutrient/water uptake, anchorage and production of gametophore. Then, specialized tip-growing cells appeared later, like pollen tubes devoted to carry the sperm cells to the ovules for seed production, and root hairs ensuring water and nutrient uptakes. Therefore, as these cell functions were spatially separated in specialized tissues, they could evolve independently and modify their cell wall structures and properties to improve their restricted functions. As such, many genes are specifically expressed in pollen including those encoding cell wall remodeling enzymes. As an example, 14 PMEs are specifically expressed in pollen (Leroux et al., 2015).

An interesting recent work using *A. thaliana* root hairs (8 μm diameter), *Torenia fournieri* pollen tubes (8 μm diameter) and *P. patens* protonemata (18-20 μm diameter) growing in microfluidic channels revealed that the three structures were able to substantially deform their tips and to grow in very narrow gaps (1 μm wide) (Yanagisawa et al., 2017). Within the three models, the tip-growing cells ceased growth when the length of the narrow gaps was too long but only pollen tubes were bursting. These data reveal that the tip of these cells are highly flexible but pollen tube tips are much more delicate due to the subtle difference of vesicle delivery rates, cell wall composition and structure. It may also suggest that the growth arrest may have triggered the sperm release as it is observed *in vivo* when the pollen tube reaches the ovule.

XyGs are detected in all gametophyte cell walls of land plants studied so far. Nevertheless, structural diversity is found among the different plant lineages, probably in relation to their distinct functions. As such, GalA-containing XyG are found in *P. patens* protonemata (reviewed by Schultink et al., 2014) and in arabidopis root hairs (Peña et al., 2012), but are not detected in angiosperm pollen tubes (Dardelle et al., 2010, 2015; Lampugnani et al., 2013) (Figure 7), suggesting a specific function of this motif in the cell wall of those water/nutrient sensing and absorbing structures.

Another interesting point is the fucosylation status of XyGs. In *A. thaliana* plants, the gametophyte pollen tubes contain higher levels of fucosylated XyGs (Figure 7) than their respective sporophyte organs (i.e., leaf) (Dardelle et al., 2010). In addition, plants that do not have fucosylated XyGs in their sporophyte cell wall organs, as Lamiids, (Figure 1), reviewed by Schultink et al. (2014), have XyGs harboring Fuc side chains in their pollen tube cell walls (Lampugnani et al., 2013; Dardelle et al., 2015). In animal cells, it was shown that Fuc residues on cell surface glycans are thought to be involved in cell-cell interactions and signaling (Li et al., 2018). We might hypothesized that these slight structural diversifications were linked to the requirement of the pollen tube to interact with the female tissues and to grow rapidly for efficient fertilization and seed setting.

Other striking differences between those gametophyte tip-growing cells are the general pattern of callose and HG depositions in protonemata, rhizoids and pollen tubes (Figure 7). Callose is almost absent in tip-growing cells of bryophytes, detected in rhizoids of *C. richardii*, transiently found at the tip and in the unique cell wall layer of gymnosperm pollen tubes, both with slow growth rates. In several gymnosperms, as *Picea sp*., and eudicots, pollen tubes started to grow faster and an increase in callose deposition in the shank of the tube is observed, while it disappeared at the pollen tube tip (Figure 7). In all the eudicots studied so far, the presence of two cell walls (inner callose-enriched layer and outer pectin-enriched layer) in the shank of the pollen tube has been a starting point of fast-growing pollen tubes. These two cell wall layers may help to counteract the circumferential turgor pressure (Figure 7). In addition, the appearance of callose plugs that are regularly deposited during growth has permitted to maintain the vegetative cell in the front of the tube and to drive the turgor pressure to the tip allowing increasing growth rates. However, while necessary for growth, no study has to our knowledge established a causal link between turgor pressure and pollen tube growth rate (Benkert et al., 1997; Kroeger et al., 2011).

The clear or less clear separation of methylesterified or non-methylesterified HG zonation is another evolution in tip-growing cells (Table 1). The presence of unesterified HGs is detected in the whole protonemal cells. In slow-growing gymnosperm pollen tubes, esterified HGs are not restricted to the tip and are found way back from the tip (Table 1; Figure 7). In fast-growing pollen tubes, a clear zonation is visible with methylesterified HGs at the tip and unesterified HGs in the sub-apical dome of the tip allowing the formation of pectate-calcium interaction, leading to cell wall stiffening. This fine regulation is promoted by PMEIs that can regulate precocious HG de-methyleterification by PMEs. As pollen tubes continue to grow at the tip, PMEIs are then endocytosed (Röckel et al., 2008), enabling PMEs to remove the methylesters present on HG. Altogether, these structural features are responsible for the so-called hard shell-soft tip or soft shell-hard core (Vogler et al., 2013), and the maintenance of turgor necessary for a fast-growth rate. Thus, modulation of pectin remodeling, callose deposition and XyG specific structural differences appear to have played fundamental roles across gametophyte tip-growth evolution. This review based on available data shows clearly that gametophyte tip-growing cells share common structural features, but differences are found.

The lack of informations is obvious on the biochemical, the distribution and the functional aspects of cell wall polymers in all the clades of the green lineages including rhizoids, pollen tubes from gymnosperms and more surprisingly in the commelinid monocots, despite the fact for the latter that major advances were performed on pollen formation using functional genomics. Surely, complementary biochemical, cell imaging and cell wall remodeling enzyme studies on these plant lineages would greatly improve our understanding on how these tip-growing cells have evolved.

This fine-tuning of the cell wall biosynthesis, deposition and enzyme remodeling is orchestrated by intracellular compartments (ER, Golgi apparatus, actin, microtubules…), and signaling components (calcium, ROS, Rho-GTPases…), that could not be treated in this review.

## Author Contributions

JD wrote the pectin section, participated to the conclusion and produced Table 1 and all the figures except Figure 1. AM wrote the callose section and participated to the introduction. LM-B and M-CK-M performed the phylogenetic analysis and produced Figure 1. AL participated in the cellulose, hemicellulose sections and the conclusion. J-CM wrote the introduction, the HRGP section, prepared Table 2 and Supplemental Table 1, participated to the conclusion and reviewed the entire manuscript. All the authors proof-read the manuscript.

### Conflict of Interest Statement

The authors declare that the research was conducted in the absence of any commercial or financial relationships that could be construed as a potential conflict of interest.
