# Supplementary material for: Evolution of Cell Wall Polymers in Tip-Growing Land Plant Gametophytes: Composition, Distribution, Functional Aspects and Their Remodeling
Source: Front Plant Sci. 2019 Apr 18;10:441. doi: 10.3389/fpls.2019.00441 (PMC6482432; doi:10.3389/fpls.2019.00441)
Supplement: Supplementary file 1 [file Table_1.pdf]

Supplemental Table 1 – List of monoclonal antibodies and reagents directed against cell wall carbohydrate epitopes.

	Probes[a]	Epitope recognized	Polymer	References
**Cellulose**	CBM3a	NA	Cellulose	Blake et al. (2006)
	CBM28	NA	Cellulose	
	Calcofluor white	NA	β-Glucan	NA
**Xylan**	LM10	[β-(1,4)-Xyl]$_5$	Xylan	McCartney et al. (2005)
	LM11	[β-(1,4)-Xyl]$_5$	Xylan/Arabinoxylan	
**Mannan**	LM21	[β-(1,4)-Man]$_{2-5}$	Mannan, glucomannan, galactomannan	Marcus et al. (2008)
	LM22	[β-(1,4)-Man]$_{2-5}$	Mannan, glucomannan	
	BS-400-4	Unknown	Galactomannan, glucomannan	Pettolino et al. (2001)
**Xyloglucan**	CCRC-M1	α-Fuc-(1,2)-β-Gal	FucogalactoXyG	Puhlmann et al. (1994)
	CCRC-M86	Unknown	XyG	Pattathil et al. (2010)
	LM15	XXXG	XyG	Marcus et al. (2008)
	LM24	XXLG/XLLG	GalactoXyG	Pedersen et al. (2012)
	LM25	XLLG/XXLG/XXXG	XyG/GalactoXyG	
**β-glucan-mix-linkage**	BS-400-3	Unknown	β-(1-3)-(1-4)-glucan	Meikle et al. (1994)
**Pectin**	JIM5	MeGalA-(1,4)-[GalA]$_4$-(1,4)-MeGalA	Weakly methylesterified HG	Clausen and Madsen (2003)
	JIM7	GalA-(1,4)-[(MeGalA)]$_4$-(1,4)-GalA	Methylesterified HG	
	LM19	[α-GalA-(1,4)]$_4$	Weakly methylesterified HG	Verhertbruggen et al. (2009)
	LM20	[α-MeGalA-(1-4)]$_4$	Methylesterified HG	
	LM5	[(1-4)-β-D-Gal]$_{>3}$	Galactan of RG-I	Jones et al. (1997)
	LM6	[(1-5)-α-L-Ara]$_{5/6}$	Arabinan of RG-I and AGP	Willats et al. (1998)
	LM8	Unknown	Xylogalacturonan	Willats et al. (2004)
	LM13	linear [(1-5)-α-L- Ara]$_5$	RH-I side chain	Moller et al. (2008)
	Propidium iodide	NA	HG	Rounds et al. (2011)
**HRGP**	JIM4	β-D-GlcA-(1,3)-α-D-GalA-(1,2)-α-L-Rha	AGP	Yates et al. (1996)
	LM2	β-linked GlcA		
	MAC207	β-GlcA-(1,3)-α-GalA-(1,2)-Rha		
	JIM8	Unknown		Pennell et al. (1991)
	JIM13	β-D-GlcA-(1,3)-α-D-GalA-(1,2)-α-L-Rha		Yates and Knox (1994); Yates et al. (1996)
	JIM14	Unknown		
	(β-Glucosyl)$_3$ Yariv (BS 100-2)	Unknown		Yariv et al. (1967)
	(β-Galactosyl)$_3$ Yariv (BS 100-8)	[β-(1,3)-Gal]$_{>5}$		Yariv et al. (1967); Kitazawa et al. (2013)
	LM1	Unknown	Extensin	Smallwood et al. (1995)
	JIM11	Unknown		Smallwood et al. (1994)
	JIM20	Unknown		
**Callose**	Aniline blue	NA	Callose	NA
	Calcofluor white	NA	β-Glucan	
	BS-400-2	β-(1,3)-glucan	Callose	Meikle et al. (1991)

[a] CCRC-M. Complex Carbohydrate Research Center-Monoclonal, LM, Leeds Monoclonal, BS, BioSupplies, JIM, John Innes Monoclonal, CBM3a. Cellulose binding module3a.
XyG. Xyloglucan, HG. Homogalacturonan, RG-I. Rhamnogalacturonan-I. AGP arabinogalactan proteins, Ara. arabinose, Fuc. fucose, Gal. galactose, GalA. galacturonic acid, Glc. glucose, GlcA. glucuronic acid, MeGalA, 6-O-methyl-galacturonate, Man. mannose, (βGlcY). (β-D-glucosyl)3 Yariv phenylglucoside, AG. Arabinogalactan, Rha. rhamnose, NA. not applicable.
For more information see the web sites http ://www.plantprobes.net/index.php, https ://www.agrisera.com/en/artiklar/plantalgal-cell-biology/cell-wall-2/index.html and http ://www.biosupplies.com.au
